# CXCR6-Deficiency Improves the Control of Pulmonary *Mycobacterium tuberculosis* and Influenza Infection Independent of T-Lymphocyte Recruitment to the Lungs

**DOI:** 10.3389/fimmu.2019.00339

**Published:** 2019-03-07

**Authors:** Anneliese S. Ashhurst, Manuela Flórido, Leon C. W. Lin, Diana Quan, Ellis Armitage, Sebastian A. Stifter, John Stambas, Warwick J. Britton

**Affiliations:** ^1^Tuberculosis Research Program Centenary Institute, The University of Sydney, Camperdown, NSW, Australia; ^2^Central Clinical School Faculty of Medicine and Health, The University of Sydney, Camperdown, NSW, Australia; ^3^School of Medicine, Deakin University, Geelong, VIC, Australia

**Keywords:** CXCR6, tuberculosis, influenza, lung, tissue-resident memory

## Abstract

T-lymphocytes are critical for protection against respiratory infections, such as *Mycobacterium tuberculosis* and influenza virus, with chemokine receptors playing an important role in directing these cells to the lungs. CXCR6 is expressed by activated T-lymphocytes and its ligand, CXCL16, is constitutively expressed by the bronchial epithelia, suggesting a role in T-lymphocyte recruitment and retention. However, it is unknown whether CXCR6 is required in responses to pulmonary infection, particularly on CD4^+^ T-lymphocytes. Analysis of CXCR6-reporter mice revealed that in naïve mice, lung leukocyte expression of CXCR6 was largely restricted to a small population of T-lymphocytes, but this population was highly upregulated after either infection. Nevertheless, pulmonary infection of CXCR6-deficient mice with *M. tuberculosis* or recombinant influenza A virus expressing P25 peptide (rIAV-P25), an I-A^b^-restricted epitope from the immunodominant mycobacterial antigen, Ag85B, demonstrated that the receptor was redundant for recruitment of T-lymphocytes to the lungs. Interestingly, CXCR6-deficiency resulted in reduced bacterial burden in the lungs 6 weeks after *M. tuberculosis* infection, and reduced weight loss after rIAV-P25 infection compared to wild type controls. This was paradoxically associated with a decrease in Th1-cytokine responses in the lung parenchyma. Adoptive transfer of P25-specific CXCR6-deficient T-lymphocytes into WT mice revealed that this functional change in Th1-cytokine production was not due to a T-lymphocyte intrinsic mechanism. Moreover, there was no reduction in the number or function of CD4^+^ and CD8^+^ tissue resident memory cells in the lungs of CXCR6-deficient mice. Although CXCR6 was not required for T-lymphocyte recruitment or retention in the lungs, CXCR6 influenced the kinetics of the inflammatory response so that deficiency led to increased host control of *M. tuberculosis* and influenza virus.

## Introduction

Pro-inflammatory chemokines play an important role in directing the recruitment and retention of lymphocytes to non-lymphoid sites after vaccination or infection (1, 2). Appreciating the contribution of chemokine receptors to pulmonary immunity is important for our understanding of protective T-lymphocyte responses against respiratory infections significant to global human health, such as *Mycobacterium tuberculosis* or influenza virus. CXCR6 (CD186), also known as Bonzo, STRL33 or TYMSTR, was originally described as a co-receptor for SIV and HIV (3); however it subsequently was found to promote homing of lymphocytes to non-lymphoid tissues (4). It is expressed on subsets of CD4^+^ and CD8^+^ T-lymphocytes where it is highly upregulated after activation, but also on subsets of natural killer (NK) and NKT cells, plasma cells, dendritic cells (DCs), innate lymphoid cells (ILCs), and MAIT cells (5–7). CXCR6 binds exclusively to the ligand CXCL16, which may exist in either a membrane bound or soluble form. Cleavage of membrane-bound CXCL16 by the proteases, ADAM-10, or ADAM-17, results in the release of soluble chemokine which acts as a chemoattractant (8). CXCL16 is expressed by activated macrophages, monocytes, DCs, B-lymphocytes, liver sinusoidal endothelial cells, and, notably, constitutively by bronchial epithelia, suggesting a role in the recruitment or long term retention of T-lymphocytes in the lungs (9–11).

Assessment of CXCR6 expression during infection or immunopathology has indicated conflicting roles for the receptor. While expression is generally upregulated at sites of inflammation, it has been associated with either disease progression or host defense, depending on the context. Association with disease progression has occurred most commonly in non-infectious settings, for example in the pathogenesis of arthritis by inducing T-lymphocyte homing to the joints and Th1 polarization (12). The role for CXCR6 during infection, however, is less clear. CXCR6^+^ cells migrate toward sites of acute bacterial tissue infection (13) and are highly upregulated on T-lymphocytes in the liver during murine *Listeria monocytogenes* infection (14). CXCR6-deficiency results in altered tissue distribution and reduced persistence of tissue resident memory (T_RM_) CD8^+^ T-lymphocytes in the liver and the skin (2, 14–16).

Within the lung, CXCR6 is more highly upregulated on human bronchoalveolar lavage (BAL)-derived and lung T-lymphocytes than those in the peripheral blood, and increased expression of CXCR6 on lung CD8^+^ T-lymphocytes correlates with disease severity in chronic obstructive pulmonary disease (17–20). CD8^+^ T-lymphocytes from human lungs with the phenotype of T_RM_ (CD69^+^ CD49a^+^ CD103^+^), are more likely to express CXCR6 than other CD8^+^ T-lymphocytes, and have increased capacity to secrete IFNγ (21–23). Further, pulmonary infection with *Pneumocystis jirovecii* is associated with significant upregulation of CXCR6 on CD4^+^ T-lymphocytes (24). Pulmonary vaccination with an adenovirus-vectored tuberculosis (TB) vaccine resulted in increased expression of CXCR6 on lung localized CD8^+^ T-lymphocytes that were associated with protection against *M. tuberculosis* (7, 25). However, the requirement for CXCR6 in the control of pulmonary infections, and in particular its influence on CD4^+^ T-lymphocyte recruitment, function, and retention in the lungs has not been established.

To examine this, we used a combination of CXCR6-reporter and gene-deficient mice to determine the expression and role of CXCR6 on CD4^+^ and CD8^+^ T-lymphocytes in the lungs of naïve mice, and in response to two different pulmonary infections. A chronic *M. tuberculosis* bacterial model was contrasted to infection with an acute respiratory virus, influenza A. A recombinant influenza A virus (rIAV) was chosen that expresses P25 (PR8-P25), the dominant I-A^b^-restricted CD4^+^ T-lymphocyte epitope derived from the *M. tuberculosis* Ag85B protein, that confers protection against *M. tuberculosis* as a mucosal vaccine (26). This enabled comparison of CD4^+^ T-lymphocytes that were specific for the same P25 epitope, but induced by either chronic bacterial infection or acute pulmonary viral infection, to determine whether CXCR6 was required for their recruitment and function and the retention of CD4^+^ T_RM_ in the lungs.

## Materials and Methods

### Mice Strains

All murine experiments were conducted with the approval of the Sydney Local Health District Animal Welfare Committee (protocols 2013/075 and 2016/044). C57BL/6 mice were obtained from Animal BioResources (Moss Vale, NSW, Australia) and all other mice were bred at the Centenary Institute. CXCR6-deficient mice (C6G) were C57BL/6 with a gene for eGFP knocked into both *Cxcr6* alleles, rendering CXCR6 non-functional (27). Breeder pairs (initially from The Jackson Laboratory) were kindly provided by Dr. Ben Roediger, Centenary Institute. Reporter mice (*Cxcr6*^+/egfp^ mice) were generated by crossing C6G with C57BL/6 mice. For littermate studies, *Cxcr6*^+/egfp^ mice were crossed to provide *Cxcr6*^+/+^ (or CXCR6^WT^) and *Cxcr6*^egfp/egfp^ (or CXCR6^KO^). Breeder pairs of P25 mice, C57BL/6 with a transgenic TCR specific for the I-A^b^ restricted Ag85B_240−254_ peptide from *M. tuberculosis* (P25) (28), were kindly provided by Joel Ernst, New York School of Medicine. C6G, and P25 mice were crossed to generate mice with P25 TCR transgenic CD4^+^ T-lymphocytes that were *Cxcr6*^egfp/egfp^ (C6GKO-P25).

### *M. tuberculosis* Culture and Infection

*M. tuberculosis* H37Rv (ATCC 27294 or BEI Resources, NIAID, NIH, NR-13648) was cultured in Middlebrook 7H9 (Difco) broth supplemented with albumin-dextrose-catalase (ADC; 10% v/v), glycerol (0.2% v/v) and Tween-80 (0.05% v/v) at 37°C. To enumerate bacteria, cultures were plated onto Middlebrook 7H10 or 7H11 (Difco) agar, supplemented with oleic-acid-albumin-dextrose catalase (OADC; 10% v/v) and glycerol (0.5% v/v), and incubated at 37°C for 21 d. Male mice were challenged with *M. tuberculosis* H37Rv by low-dose aerosol infection (100 CFU) in an inhalation exposure system (Glas-Col, Terre Haute, IN). Bacterial loads in the lungs, spleen, or liver were determined by plating serial dilutions of the tissue homogenate onto 7H10 or 7H11 plates, incubated at 37°C for ~21 d.

### Recombinant Influenza A Viral Infection

Recombinant H1N1 IAV PR8 (A/Puerto Rico/8/1934) expressing the P25 epitope of Ag85B from *M. tuberculosis* (PR8-P25) was prepared as previously described (26). Female mice were anesthetized with ketamine/xylazine (50/6.25 mg/kg) by intra-peritoneal injection and 20 PFU was delivered intra-nasally in 50 μl PBS.

### Organ Collection and Processing

Mice were sacrificed by CO_2_ asphyxiation and tissues removed aseptically. Mice were injected i.v 3 min prior to euthanasia with anti-CD45-APCCy7 (5 μg; BD Biosciences, CA) to distinguish labeled vascular leukocytes from unlabeled lung parenchymal cells (29). BAL were obtained by inflating the lungs with 1 ml PBS and collecting the fluid to isolate the cells. The post-caval lung lobe was collected into 10% neutral buffered formalin, paraffin embedded and processed for hematoxylin and eosin staining using standard techniques. For isolation of lung leukocytes, lung tissue in complete RPMI media [L-glutamine and 25 mM Hepes (Invitrogen, CA), FCS (10% v/v), 2-mercaptoethanol (50 μM; Sigma, MO) and PenStrep (100 U/ml; Invitrogen)] was digested with collagenase type 4 (50 U/ml; Sigma) and DNAse I (13 μg/ml; Sigma) at 37°C for 45 min prior to homogenization and multiple filtration steps. Lymph nodes and spleens were filtered (70 μm) in complete RPMI and the leukocytes pelleted by centrifugation (500 *g*). Erythrocytes were removed by ACK lysis buffer.

### Flow Cytometry

P25-specific T-lymphocytes were labeled with Ag85_240−254_-loaded I-A^b^ tetramer-PE (*M*. *tuberculosis* peptide sequence: FQDAYNAAGGHNAVF; National Institute of Health Tetramer Core Facility, Emory University Vaccine Center, Atlanta, GA) for 1 h at 37°C. Antigen-specific cytokine production by murine T-lymphocytes was enumerated by peptide stimulation and intra-cellular immunostaining (ICS). Peptides utilized were P25 (I-A^b^ restricted CD4^+^ T-lymphocyte epitope, Ag85B_240−254_, from *M. tuberculosis*), TB10.4_3−11_ (H-2K^b^-restricted CD8^+^ T-lymphocyte epitope from *M. tuberculosis*) and NP_366−375_ (H-2D^b^ restricted immunodominant CD8^+^ T-lymphocyte from IAV nucleoprotein). Up to 4 × 10^6^ lymphocytes were stimulated for 1–4 h (37°C, 5% CO_2_) with appropriate peptide (5–10 μg/ml; Genscript, NJ) and further incubated (3–16 h, 37°C, 5% CO_2_) with Brefeldin A (10 μg/ml; Sigma) to allow intracellular accumulation of cytokine prior to immunostaining. Single-cell suspensions were incubated with anti-mouse CD16/CD32 (2.4G2; BD Biosciences) in FACS wash (PBS with 2% FCS) to block Fc receptors and then with antibody mix to label surface markers. The cells were fixed (BD Cytofix/perm) and washed (BD Perm/Wash), then intra-cellular markers stained with appropriate antibody mix (in BD Perm/Wash). Antibodies included the following, from BD Pharmingen, anti-CD8-PB (clone 53-6.7), anti-CD4-AF700 (RM4-5), anti-CD4-PECy7 (RM4-5), anti-CD45-APCCy7 (30-F11), anti-CD69-FITC (H1.2F3), anti-CD69-PE (H1.2F3), anti-CD62L-PE (MEL-14), anti-CD11b-AF700 (M1/70), anti-NK1.1-PE (PK136), anti-CD8-PerCP (53-6.7), anti-Vβ11-PE (RR3-15), anti-CD45.1-APC (A20), anti-CD45.2-Biotin (104), anti-CD45.1-Biotin (A20), anti-CD11a-BV510 (M17/4); anti-KLRG-1-PECy7 (2F1; eBioscience, CA); from Biolegend (CA), anti-CD69-BV785 (H1.2F3), anti-CD4-AF700 (GK1.5), anti-CD103-PerCPCy5.5 (2E7), anti-CD3-PECy7 (145-2C11), anti-CD44-PECy7 (IM7), anti-CXCR3-APC (CXCR3-173), anti-CD11a-biotin (M17/4), anti-Ly6G-PerCPCy5.5 (1A8), anti-B220-APC (RA3-6B2), anti-CD45.2-PB (104), anti-IFNγ-APC (XMG1.2), anti-IL-17A-PB (TC11-18H10.1), anti-TNFα-PE (MP6-XT22), anti-CD45.1-PB (A20), and from Invitrogen, live/dead fixable blue dead cell stain and streptavidin-PO. The data were acquired using an LSRFortessa (BD) and analyzed with FlowJo (Tree Star Inc.).

### CD4^+^ Transgenic T-Lymphocyte Isolation and Adoptive Transfer

Spleens were harvested from TCR-Tg P25 or C6GKO-P25 mice and processed to single cell suspensions. CD4^+^ T-lymphocytes were enriched via negative selection using the EasySep Mouse CD4^+^ T-cell isolation kit (STEMCELL Technologies, Vancouver), according to manufacturer's instructions. Purification efficiency was assessed by flow cytometry (usually ≥90%), and the proportion of Vβ11 transgenic TCR^+^ CD4^+^ CD45.1^+^ cells was determined. Cells were washed, resuspended in sterile PBS and the concentration standardized such that 5 × 10^4^ CD45.1^+^ CD4^+^ TCR-transgenic T-lymphocytes were transferred to recipient C57BL/6 mice by i.v injection 1 day prior to PR8-P25 infection.

### RNA Extraction From Isolated Cells or Murine Lungs and qPCR

Lung or spleen cell suspensions from mice 6 weeks after PR8-P25 infection were pooled. P25-specific CD45.1^+^ CD4^+^ T-lymphocytes were enriched via negative selection as above, then sorted in a BD FACSAria II into: lung effector memory (L-EM, CD69^−^CD62L^−^), lung resident memory (L-RM, CD69^+^), spleen effector memory (S-EM, CD69^−^CD62L^−^), or spleen central memory (S-CM, CD69^−^CD62L^+^) as previously described (30). Gating strategies may be viewed in Supplementary Figures 1,2. Effector P25 cells (S-eff) were obtained from mice 11 days p.i. RNA was extracted from sorted cells according to manufacturer's instructions (QIAGEN RNeasy Mini). For analysis of mRNA from whole lungs, the post-caval lung lobe of mice was immersed in RNA*later* (Sigma) for 16 h (4°C) prior to storage at −80°C. The lung lobe was suspended in 1 ml TRIsure (Bioline) with a stainless steel bead (Qiagen, Germany) and homogenized by bead beating (Qiagen TissueLyser LT). RNA was purified using TRIsure (Bioline) as per manufacturer's instructions, and concentration and purity established by spectrophotometry (Nanodrop 2000; Thermo Fisher Scientific). DNase I (NEB) digestion was conducted and RNA was reverse transcribed using a Tetro cDNA synthesis kit with random primers according to manufacturer's instructions (Bioline). PCR was used to amplify *GAPDH* from cDNA to confirm synthesis, achieved with MyTaq Red DNA polymerase (Bioline) as per manufacturer's instructions in a Labnet Multigene Gradient Thermal Cycler (annealing temperature 57°C) with the primers CATGGCCTTCCGTGTTCCTA and GCGGCACGTCAGATCCA (5′-3′; Sigma). The PCR product was assessed by agarose gel electrophoresis. cDNA was prepared for qPCR with SensiFAST SYBR master mix (Bioline) and primers (200–400 nM; Sigma): *18S* Fw GTAACCCGTTGAACCCCATT or TACACCAGCAGCAGGATCAG, Rv CCATCCAATCGGTAGTAGCG, *Cxcr6* Fw TAGTGGCTGTGTTCCTGCTG, Rv GGCAGCCGATATCCTTCATA, and *IAV NP* Fw CAGCCTAATCAGACCAAATG, Rv TACCTGCTTCTCAGTTCAAG. The reaction was carried out using a LightCycler 480 II (Roche) or Bio-Rad CFX96. The threshold cycle of individual genes was normalized to the value of 18 s rRNA (ΔC_T_) for each sorted cell population, or for total lung RNA as fold increases over that in WT controls, and gene expression was calculated by the 2^(−ΔCT)^ method. For absolute viral nucleoprotein (NP) quantification, a standard curve was generated to determine absolute viral NP mRNA copy number among sample mRNA, as previously described (31).

### Statistical Analysis and Data Availability

Statistical analysis was performed using GraphPad Prism 6 or 7 software (GraphPad Software, La Jolla, CA). Differences between two groups were analyzed by Student's *t*-test, or between multiple groups by ANOVA with Bonferroni *post-hoc* comparison, and were considered significant when the *P*-values were ≤0.05. The raw data supporting the conclusions of this manuscript will be made available by the authors, without undue reservation, to any qualified researcher.

## Results

### Expression of CXCR6 in the Lungs of Naïve or Infected Mice

**Figure 1:**
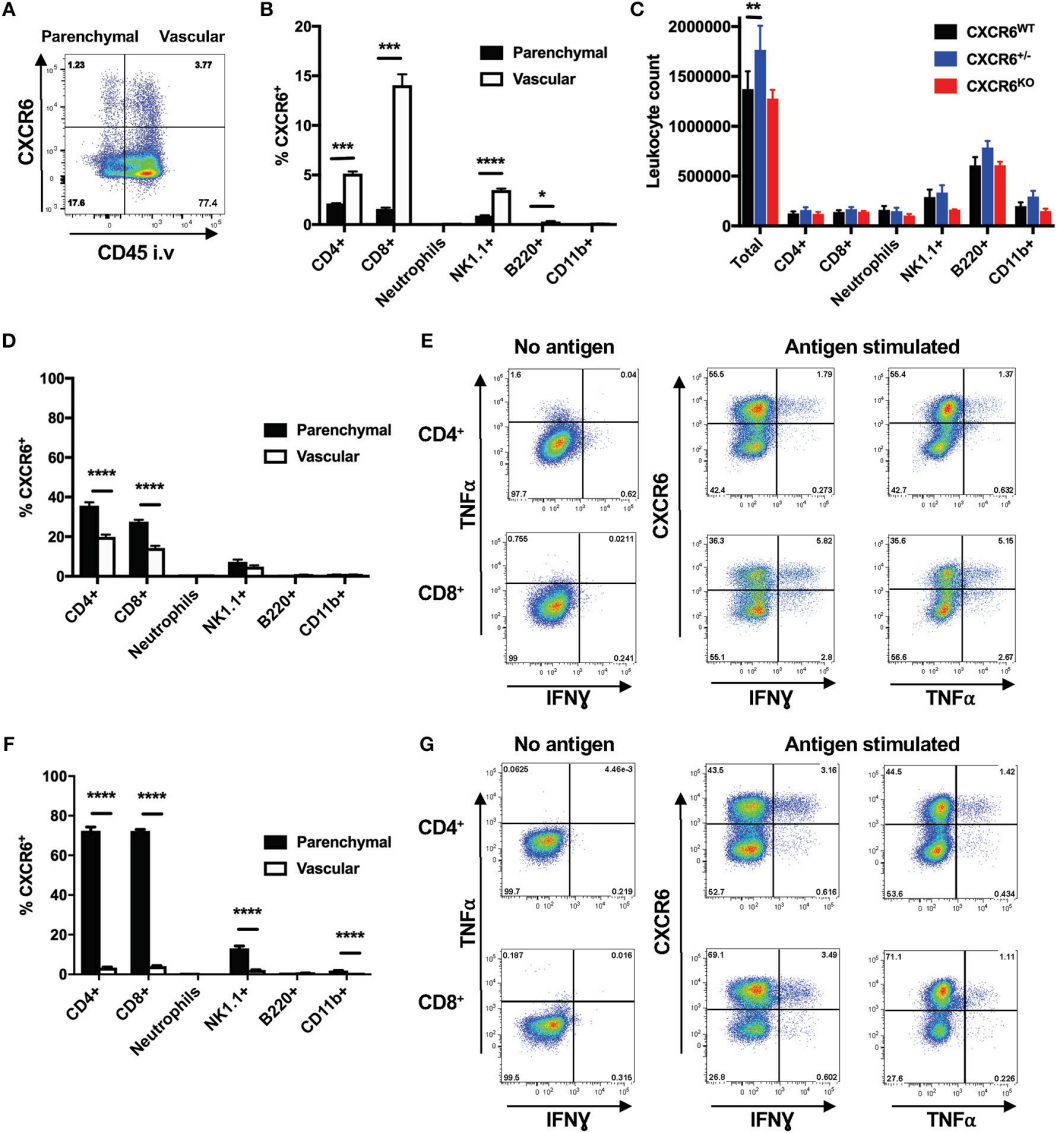
Expression pattern of CXCR6 in the lungs of mice. CXCR6-reporter mice were injected i.v with anti-CD45 antibody to label intra-vascular lung leukocytes immediately prior to collection of lung cells and analysis by flow cytometry. **(A)** CXCR6^−^ or CXCR6^+^ leukocyte populations in the lung parenchyma (CD45^−^) or vasculature (CD45^+^) of naïve mice. **(B)** Percentage of leukocyte populations in naïve lung parenchyma or vasculature expressing CXCR6 (*n* = 3). **(C)** Enumeration of different leukocyte populations in the lungs of naïve CXCR6^WT^, CXCR6^+/−^, and CXCR6^KO^ mice (*n* = 3). **(D,E)** CXCR6-reporter mice (*n* = 5) were infected with *M. tuberculosis* (~100 CFU) for 6 weeks. **(D)** Percentage of different leukocyte populations in the lung parenchyma or vasculature expressing CXCR6. **(E)** Lymphocytes from the lungs were recalled with peptide antigen, or as a control were cultured in the same manner but without antigen, and the expression of CXCR6 by parenchymal cytokine-secreting P25-specific CD4^+^ and TB10.4_3−11_-specific CD8^+^ T-lymphocytes was determined. **(F,G)** CXCR6-reporter mice (*n* = 4) were infected i.n with PR8-P25. **(F)** Percentage of leukocyte populations in the lung parenchyma or vasculature expressing CXCR6 at day 10. **(G)** Lymphocytes from the lungs at day 20 were recalled with peptide antigen, or as a control were cultured in the same manner but without antigen, and the expression of CXCR6 by parenchymal cytokine-secreting P25-specific CD4^+^ and NP_366−375_-specific CD8^+^ T-lymphocytes was determined. Data are the means ± SEM and are representative of repeat experiments. The statistical significance of differences were analyzed by **(B,D,F)** multiple *t*-tests with correction for multiple comparisons using the Holm-Sidak method or **(C)** by ANOVA with Dunnett's multiple comparisons test (^*^p < 0.05, ^**^p < 0.01, ^***^p < 0.001, ^****^p < 0.0001).

Reporter mice (CXCR6^+/egfp^) were used to assess the expression of CXCR6, and this was detected on ~5% of all lung leukocytes in naïve mice. CXCR6 did not preferentially define leukocytes of the lung parenchyma or vasculature. In addition, the majority of leukocytes were located in the vasculature (Figure 1A). In naïve mice, a greater proportion of vascular lymphocytes and NK1.1^+^ cells expressed CXCR6 compared to their parenchymal counterparts. In accordance with previously published observations, neutrophils, B-cells and CD11b^+^ cells did not express appreciable levels of CXCR6 (Figure 1B). To determine if the absence of CXCR6 impacted the abundance of leukocyte populations in the lungs in a naïve setting, select major subsets were enumerated in CXCR6^WT^, CXCR6-reporter, or CXCR6^KO^ mice. There was no change in CD4^+^ or CD8^+^ T-lymphocyte populations, and while there was some reduction in the neutrophil and NK1.1^+^ populations of naïve CXCR6^KO^ mice, this was minimal and did not reach statistical significance (Figure 1C).

To examine changes in CXCR6 expression following infection, CXCR6^+^ populations were examined 6 weeks after *M. tuberculosis* infection, when lung T-lymphocyte responses are well established. There was a large increase in the proportion of leukocytes located within the lung parenchyma (data not shown), and CXCR6^+^ cells were present in both the lung parenchyma and vasculature. A large influx of CD8^+^ and CD4^+^ T-lymphocytes was seen after 6 weeks, and in contrast to naïve mice a substantial proportion of the parenchymal T-lymphocytes was CXCR6^+^ (Figure 1D). This was also seen at 3 and 12 weeks after infection (Supplementary Figures 3A–D). To see if CXCR6 expression correlated with the ability of lymphocytes to express cytokines, leukocytes from the lungs were stimulated with *M. tuberculosis* CD4^+^ and CD8^+^ T cell epitopes, P25 or TB10.4_3−11_, and T-lymphocyte co-expression of CXCR6 and cytokines examined. In line with the increased localization of CXCR6^+^ cells to the lung parenchyma, and their activation by antigen presenting cells at this site, we observed higher production of IFNγ (CD4^+^ *p* < 0.0001, CD8^+^ *p* < 0.001), and TNFα (CD4^+^ *p* < 0.001, CD8^+^ *p* < 0.001) by CXCR6^+^ compared to CXCR6^−^ T-lymphocytes (Figure 1E), although cytokine production could be observed by both populations, particularly for CD8^+^ T-lymphocytes.

These responses were contrasted to those induced by an acute pulmonary infection, using the PR8-P25 virus. This also provided an opportunity to examine the generation of CD4^+^ T-lymphocytes in the lungs specific to the same P25 epitope as in *M. tuberculosis*, but in a different inflammatory context of acute viral infection. Lung leukocytes were examined at day 10 after PR8-P25 infection, when the peak T-lymphocyte response to influenza occurs (31). As observed with *M. tuberculosis* infection, there was a large increase in the proportion of leukocytes located within the lung parenchyma (data not shown), and this included the majority of the CXCR6^+^ cells. Nearly 80% of T-lymphocyte populations in the parenchyma expressed CXCR6, as did ~18% of lung parenchymal NK1.1^+^ cells (Figure 1F). The proportion of parenchymal CXCR6^+^ T-lymphocytes remained elevated at day 20, and slowly waned until returning to levels similar to baseline by day 42 (Supplementary Figures 3E–H). To examine if CXCR6 expression correlated with the ability of lymphocytes to express cytokines in this acute infection model, leukocytes from the lungs at day 20 after infection were stimulated with the encoded CD4^+^ T cell epitope, P25, or the endogenous CD8^+^ T cell epitope, NP_366−375_, and similarly to *M. tuberculosis* infection, production of IFNγ (CD4^+^ *p* < 0.01, CD8^+^ *p* < 0.01) and TNFα (CD4^+^ *p* < 0.05, CD8^+^ *p* < 0.01) correlated with CXCR6 expression (Figure 1G). Therefore, CXCR6 is expressed at a low basal level in the lungs of naïve mice, primarily on T-lymphocyte populations, but this is highly upregulated during infection and is associated with co-expression of inflammatory cytokines.

### Resistance of CXCR6-Deficient Mice to *M. tuberculosis* Challenge

**Figure 2:**
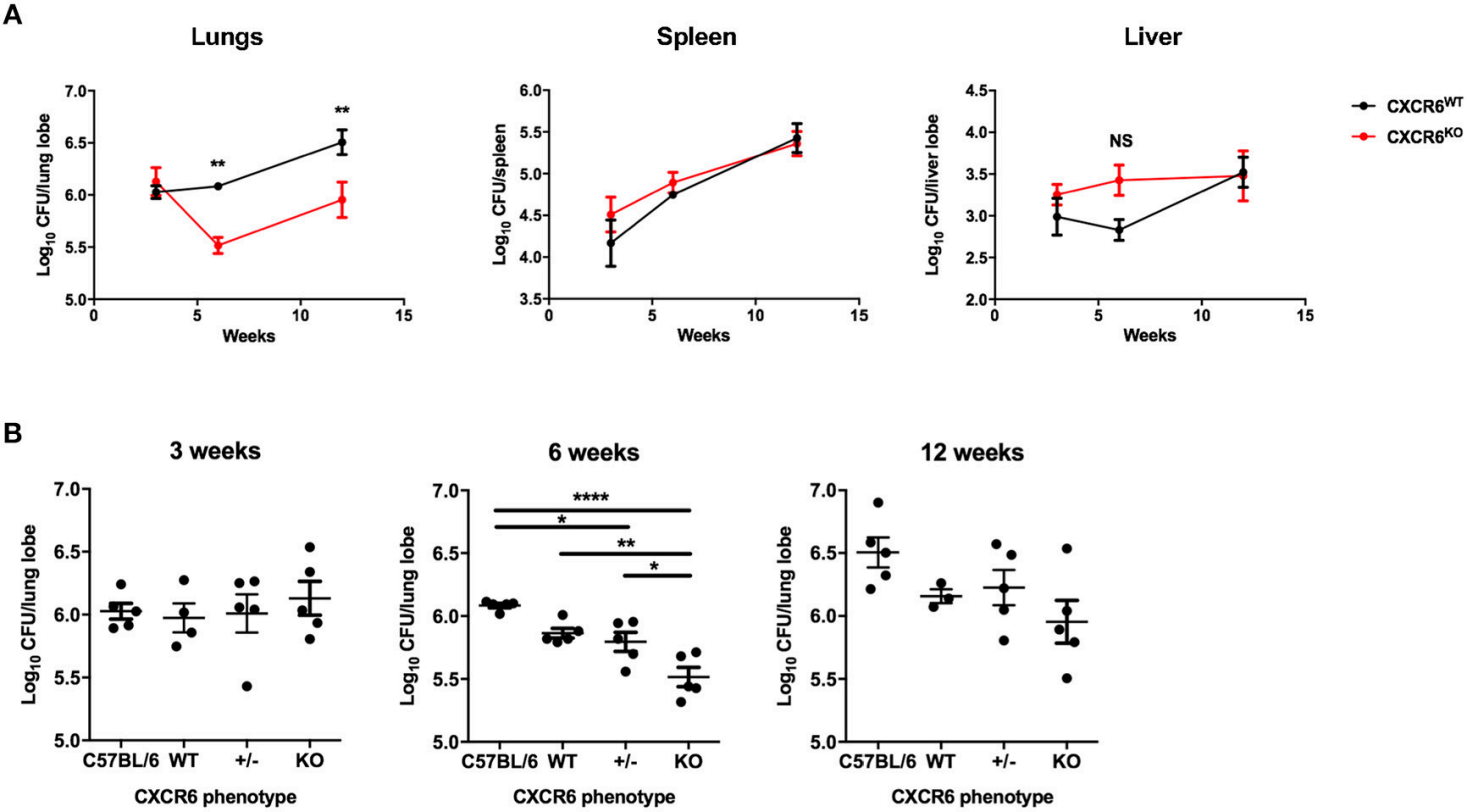
CXCR6-deficient mice have improved control of *M. tuberculosis* infection in the lungs. **(A)** CXCR6^WT^ (C57BL/6, black line) or CXCR6^KO^ (red line) mice (*n* = 5) were infected with *M. tuberculosis* (~100 CFU) by aerosol, and the kinetics of bacterial growth in the lungs, spleen, and liver were determined at 3, 6, and 12 weeks. **(B)** C57BL/6 and littermate matched CXCR6^WT^, CXCR6^+/−^, and CXCR6^KO^ mice (*n* = 3–5) were similarly infected and bacterial counts enumerated in the lungs at 3, 6, and 12 weeks. Data are the means ± SEM and are representative of four experiments. The statistical significance of differences between groups and WT controls were analyzed by ANOVA with Bonferroni *post-hoc* comparison (^*^p < 0.05, ^**^p < 0.01, ^****^p < 0.0001, NS, not significant).

To assess whether CXCR6 is required for resistance to *M. tuberculosis*, wild type (C57BL/6) or CXCR6-deficient mice were infected with a low dose of virulent *M. tuberculosis* by aerosol. The bacterial load in the lungs, spleen, and liver was enumerated at 3, 6, and 12 weeks post infection (p.i). CXCR6-deficient mice had no change to their bacterial burden at the early time point of 3 weeks p.i. However, by 6 weeks p.i, CXCR6-deficient mice had a significant reduction (*p* < 0.01) in lung bacterial burden. There was also a trend for reduced bacterial burden at 12 weeks that was significant in some, but not all, experiments (Figure 2A; *p* < 0.01). There were no significant differences between the systemic bacterial burden in the spleen or liver at any time point (Figure 2A). To verify that these differences were not due to minor strain variations, C57BL/6 and littermate-matched CXCR6^WT^, CXCR6^+/−^, and CXCR6^KO^ mice were infected with *M. tuberculosis* and similarly, a consistent, significant reduction in the lung bacterial load of CXCR6^KO^ mice was seen at 6 weeks (*p* < 0.0001 compared to C57BL/6, *p* < 0.01 compared to CXCR6^WT^; Figure 2B). No pathological changes were observed between CXCR6^WT^ and CXCR6^KO^ mice in hematoxylin and eosin stained lung sections (Supplementary Figure 4). Therefore, CXCR6-deficiency was advantageous for the control of *M. tuberculosis* in the lungs at chronic time points.

### Leukocyte Recruitment and Phenotype in *M. tuberculosis* Infection of CXCR6-Deficient Mice

**Figure 3:**
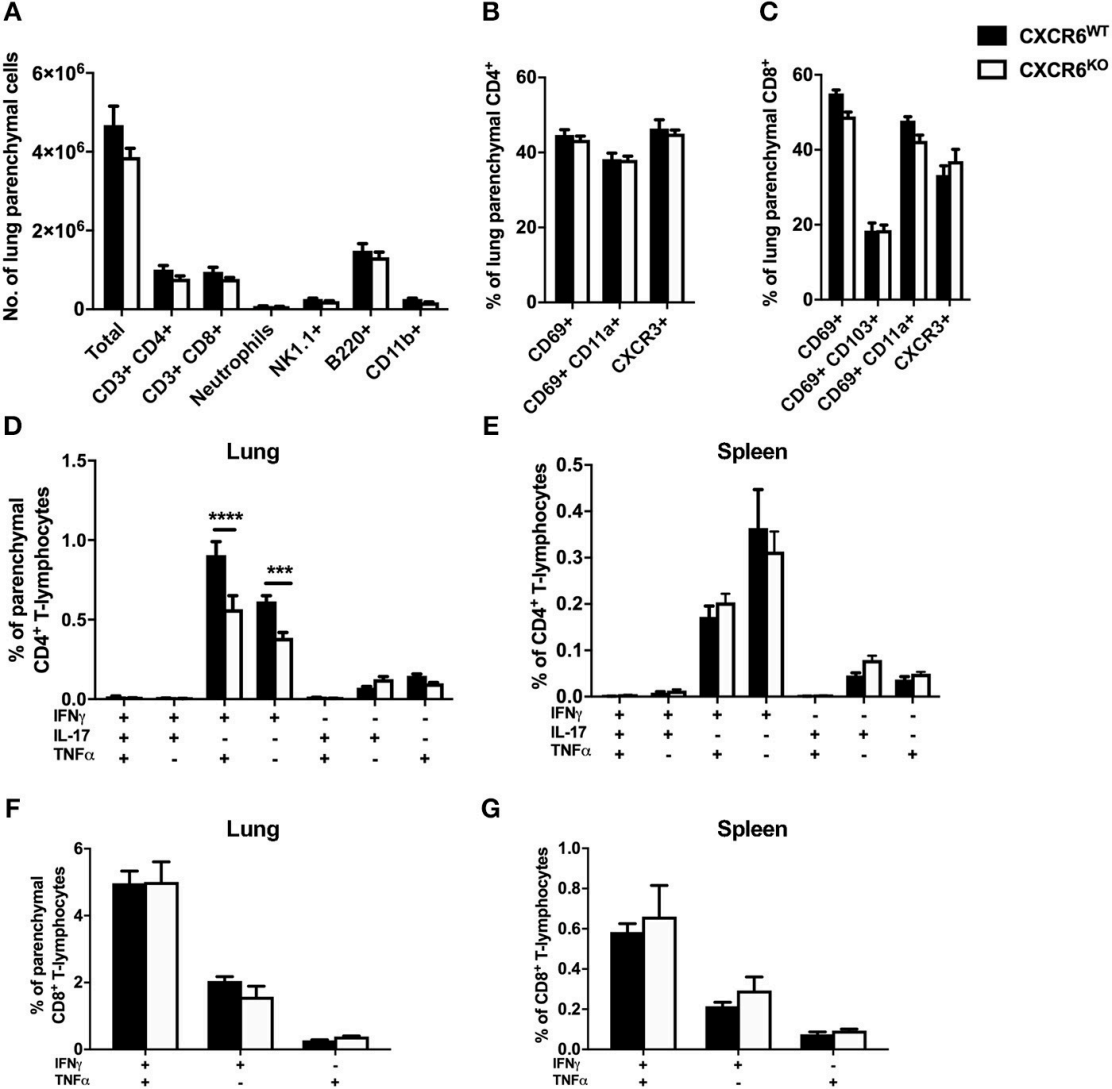
CXCR6-deficient mice have reduced Th1 T-lymphocyte responses in the lung parenchyma without changes to leukocyte recruitment at 6 weeks of *M. tuberculosis* infection. CXCR6^WT^ (C57BL6; closed bars) or CXCR6^KO^ (open bars) mice (*n* = 5) were infected with *M. tuberculosis* by aerosol and at 6 weeks were injected i.v with anti-CD45 antibody to label intra-vascular leukocytes. **(A)** Leukocyte recruitment to the lung parenchyma (CD45^−^) was enumerated by flow cytometry. Expression of surface activation markers and CXCR3 was assessed on **(B)** CD4^+^ and **(C)** CD8^+^ T-lymphocytes. Leukocytes were stimulated with peptide followed by ICS and flow cytometry. P25-specific CD4^+^ T-lymphocyte cytokine expression in the **(D)** lung parenchyma (CD45^−^) and **(E)** spleen. TB10.4_3−11_-specific CD8^+^ T-lymphocyte cytokine expression in the **(F)** lung parenchyma (CD45^−^) and **(G)** spleen. Data are the means ± SEM and are representative of three repeat experiments. The statistical significance of differences between groups were analyzed by ANOVA with Bonferroni *post-hoc* comparison (^***^p < 0.001, ^****^p < 0.0001).

Recently, it has been recognized that T-lymphocytes which localize to the lung parenchymal environment, rather than the vasculature, are responsible for protective immunity in *M. tuberculosis* infection (30, 32). Therefore, to assess whether CXCR6 was required for the development of effector responses and recruitment to the lung parenchyma, leukocytes from mice 3, 6, and 12 weeks after *M. tuberculosis* infection were analyzed by flow cytometry. The data are shown at 6 weeks p.i when there was a decrease in the lung bacterial burden in CXCR6^KO^ mice (Figure 3A). There were no significant differences in the number of total leukocytes and CD4^+^ and CD8^+^ T-lymphocytes in the lungs at 6 weeks p.i (Figure 3A) or at 3 or 12 weeks (data not shown). Furthermore, there were no differences between the percentage and total number of any of the non-T-lymphocyte leukocyte subsets, including neutrophils, NK1.1^+^ cells, B-lymphocytes, and CD11b^+^ cells (Figure 3A and data not shown). There was no difference to the expression of surface activation and homing markers, such as CD69, CD11a, and CXCR3 on either CD4^+^ or CD8^+^ T-lymphocytes (Figures 3B,C). Given the strong correlation between cytokine expression and CXCR6^+^ lymphocytes observed earlier (Figure 1E), we next evaluated whether cytokine expression was affected in the absence of CXCR6. At 6 weeks, CXCR6-deficient mice had significantly reduced Th1-type cytokine production by P25-specific CD4^+^ T-lymphocytes in the lung parenchyma, particularly IFNγ^+^TNFα^+^ (*p* < 0.0001) and IFNγ^+^ (*p* < 0.001) populations (Figure 3D). This difference was unique to the lung parenchyma and was not present in the spleen (Figure 3E) or in the lungs at 3 or 12 weeks. Interestingly, in contrast to P25-specific CD4^+^ T-lymphocytes, there was no reduction in cytokine production by TB10.4_3−11_-specific CD8^+^ T-lymphocytes in the lung parenchyma (Figure 3F) or spleen (Figure 3G) of CXCR6-deficient mice at any time point. Therefore, CXCR6 was not required for the recruitment of leukocytes to the lungs during *M. tuberculosis* infection, but CXCR6 deficiency was associated with reduced P25-specific Th1 responses in the lung parenchyma.

### Resistance of CXCR6-Deficient Mice to Influenza Challenge

**Figure 4:**
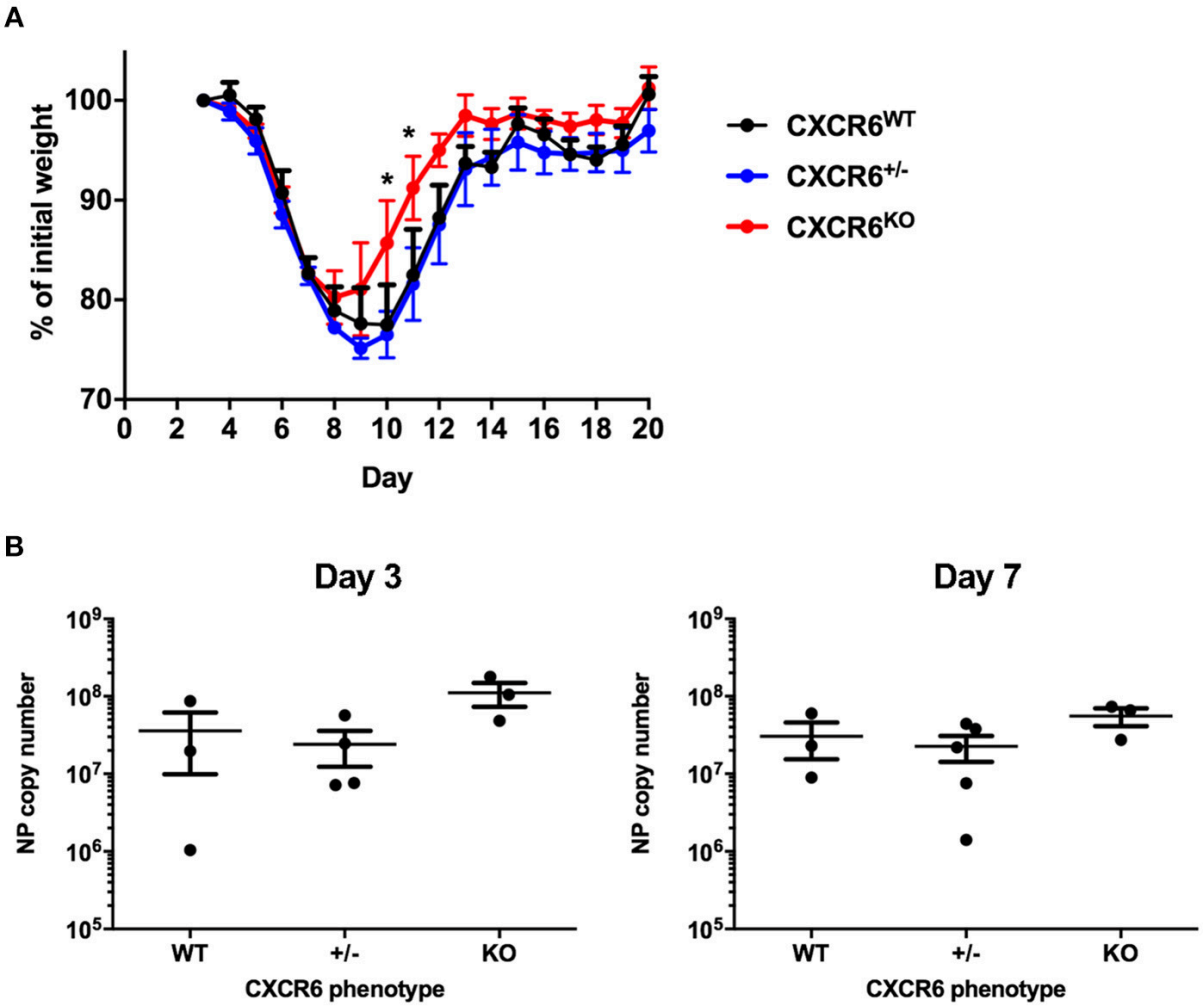
CXCR6-deficient mice experience reduced weight loss after acute influenza infection without change in viral load. **(A)** Comparison of weight loss after i.n PR8-P25 infection between CXCR6^WT^, CXCR6^+/−^, CXCR6^KO^ littermates, as a percentage of weight for individual mice from day 3 (*n* = 4–5). **(B)** NP viral copy number was enumerated in the lungs at days 3 and 7 after infection by RT-PCR in littermates (*n* = 3–5). Data are the means ± SEM and are representative of repeat experiments. The statistical significance of differences between groups were analyzed by ANOVA with Bonferroni *post-hoc* comparison to WT controls (^*^p < 0.05).

To assess whether the phenotypes of reduced disease severity (Figure 2) and reduced lung Th1-cytokine responses (Figure 3D) seen in CXCR6-deficient mice were specific to chronic bacterial infection with *M. tuberculosis*, we also examined the requirement of CXCR6 for resistance to an acute viral challenge with influenza A, which generates a substantial and contrasting inflammatory response in the lungs. WT, CXCR6-heterozygous, or CXCR6-deficient littermate-matched mice were infected i.n with PR8-P25 and their body weight monitored. Infection of WT C57BL/6 mice with PR8 IAV usually results in up to 30% weight loss, peaking at ~10 days p.i (26, 33). Surprisingly, in the absence of CXCR6, mice with acute rIAV lung infection had significantly less systemic effects, demonstrated by reduced weight loss compared to WT mice (*p* < 0.05; Figure 4A) and by earlier recovery. Survival of WT, CXCR6-heterozygous, and CXCR6-deficient mice were equivalent and was 100% in the majority of experiments. To examine if the earlier recovery of CXCR6-deficient mice was a result of decreased viral burden, we enumerated viral load by NP copy number in the lungs of infected animals at day 3 and 7 p.i. There were no significant differences in viral copy number between groups at either time point, suggesting that the reduced weight loss and earlier recovery of CXCR6^KO^ mice was not due to altered pathogen burden (Figure 4B). No pathological changes were observed between CXCR6^WT^ and CXCR6^KO^ mice in hematoxylin and eosin stained lung sections (Supplementary Figure 5).

### Leukocyte Recruitment and Phenotype in Influenza Infection of CXCR6-Deficient Mice

**Figure 5:**
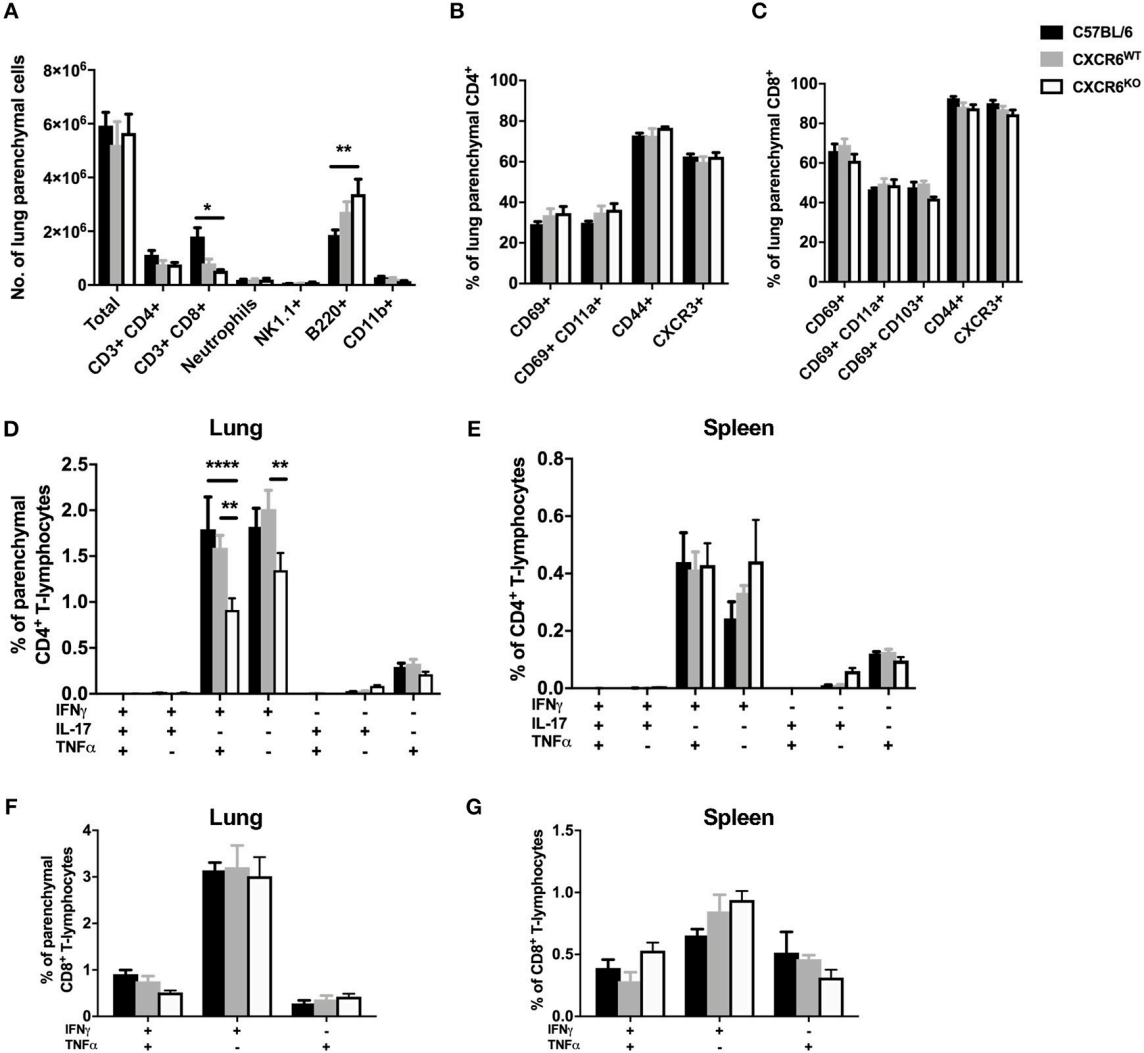
CXCR6-deficient mice have reduced Th1 T-lymphocyte responses in the lung parenchyma at day 20 after influenza infection without changes to leukocyte recruitment. C57BL/6 (closed bars), CXCR6^WT^ (gray bars), or CXCR6^KO^ (open bars) mice (*n* = 4) were infected with PR8-P25 and at 20 days were injected i.v with anti-CD45 antibody to label intra-vascular leukocytes. **(A)** Leukocyte recruitment to the lung parenchyma (CD45^−^) was enumerated by flow cytometry. Expression of surface activation markers and CXCR3 was assessed on **(B)** CD4^+^ and **(C)** CD8^+^ T-lymphocytes. Leukocytes were stimulated with peptide followed by ICS and flow cytometry. P25-specific CD4^+^ T-lymphocyte cytokine expression in the **(D)** lung parenchyma (CD45^−^) and **(E)** spleen. NP_366−375_-specific CD8^+^ T-lymphocyte cytokine expression in the **(F)** lung parenchyma (CD45^−^) and **(G)** spleen. Data are the means ± SEM and are representative of repeat experiments. The statistical significance of differences were analyzed by ANOVA with Bonferroni *post-hoc* comparison (^*^p < 0.05, ^**^p < 0.01, ^****^p < 0.0001).

To determine the requirement for CXCR6 in the development of protective T-lymphocyte responses and recruitment to the lung parenchyma in the context of acute pulmonary infection, C57BL/6 or CXCR6^WT^, and CXCR6^KO^ littermates were infected with PR8-P25 and at day 20, following clearance of the virus, leukocyte recruitment was assessed by flow cytometry. There were no significant differences in the total leukocyte or CD4^+^ T-lymphocyte count in the lungs; however, there was a small reduction in CD8^+^ T-lymphocytes between C57BL/6 and CXCR6^KO^ mice (*p* < 0.05; Figure 5A). There were no consistent significant differences in the expression of surface activation markers, including CD69, CD11a, CD103, CD44, and CXCR3, on either CD4^+^ or CD8^+^ T-lymphocytes (Figures 5B,C). Of note, CXCR3 was expressed on the majority of T-lymphocytes in the lung parenchyma, particularly CD8^+^ cells. As observed during *M. tuberculosis* infection, CXCR6^KO^ mice had significantly reduced Th1-type cytokine production by P25-specific CD4^+^ T-lymphocytes in the lung parenchyma, particularly IFNγ^+^TNFα^+^ and IFNγ^+^ populations (*p* < 0.01; Figure 5D). This difference was restricted to the lung parenchyma and there were no significant differences between groups in the spleen (Figure 5E). In contrast to CD4^+^ T-lymphocytes, there was no decrease in cytokine production by NP-specific CD8^+^ T-lymphocytes in the lung parenchyma (Figure 5F) or spleen (Figure 5G). Therefore, as in the case of *M. tuberculosis* infection, CXCR6 was not required for leukocyte recruitment in response to rIAV, but CXCR6-deficiency led to reduced Th1 responses in the lung parenchyma.

### Effect of CXCR6-Deficiency on Recruitment of Antigen-Specific CD4^+^ T-Lymphocytes

**Figure 6:**
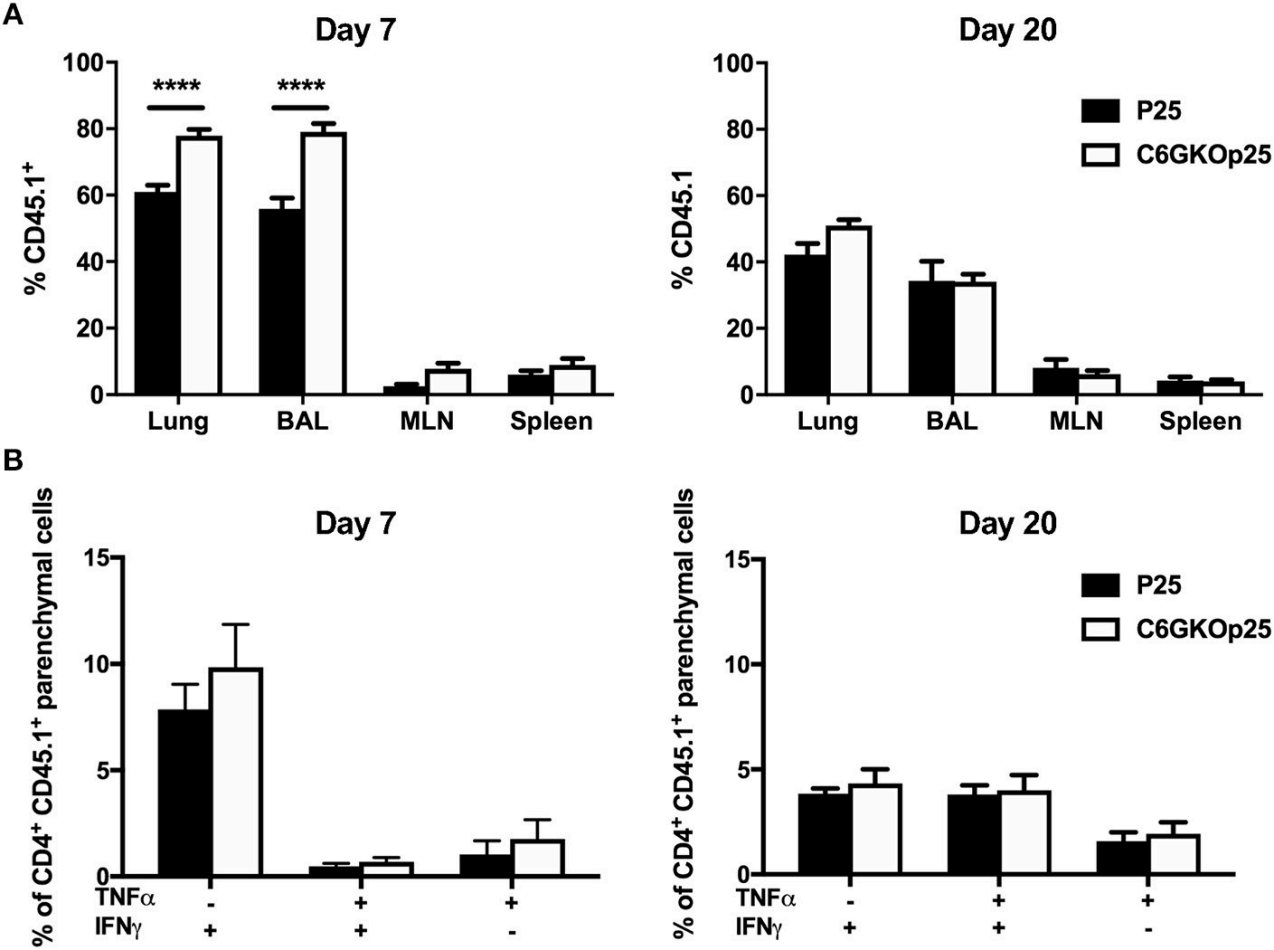
CXCR6-deficient CD4^+^ T-lymphocytes are recruited to the lung parenchyma and airways more rapidly than WT cells, but have comparable Th1-type cytokine responses. Female C57BL/6 mice (*n* = 4) received 5 × 10^4^ wild type (P25) or CXCR6^KO^ P25-specific (C6GKO-P25) CD45.1^+^ CD4^+^ T-lymphocytes by adoptive transfer and 1 day later were infected i.n with PR8-P25. At 7 or 20 days, infected mice were injected i.v with anti-CD45 antibody to label intra-vascular leukocytes. **(A)** Proportion of total CD4^+^ T-lymphocytes in the lung parenchyma, BAL, MLN, and spleen that were CD45.1^+^. **(B)** Lung leukocytes were stimulated with P25 peptide and cytokine expression assessed in CD4^+^ CD45.1^+^ lung parenchymal cells by intra-cellular staining. Data are the means ± SEM and are representative of repeat experiments. The statistical significance of differences between groups were analyzed by ANOVA with Bonferroni *post-hoc* comparison (^****^p < 0.0001).

To determine whether the altered Th1 phenotype observed after infection in the lungs of CXCR6-deficient mice was due to an intrinsic defect in CXCR6^KO^ CD4^+^ T-lymphocytes, CXCR6^KO^ P25 TCR transgenic mice were generated on a CD45.1 background (C6GKO-P25). WT P25 or C6GKO-P25 CD45.1^+^ CD4^+^ T-lymphocytes were isolated and adoptively transferred into CD45.2 C57BL/6 recipients, and the mice infected with PR8-P25. At day 7 p.i, a substantial population of the transferred lymphocytes could be identified in the lung parenchyma, BAL, mediastinal lymph node (MLN), and spleen. Of the total CD4^+^ T-lymphocytes detected within the lung parenchyma, a greater proportion of these were CD45.1^+^ in mice receiving C6GKO-P25 cells than P25 wild type cells (*p* < 0.0001). A similar observation was seen in the BAL (*p* < 0.0001); however, there were similar proportions of CD45.1^+^ T-cells between groups within the MLN and spleen (Figure 6A). By day 20, there were no significant differences in the proportion of CD45.1^+^ cells in the lung parenchyma, BAL, MLN, or spleen, although there was a trend toward increased C6GKO-P25 cells in the lung parenchyma (Figure 6A). There was no decrease in the capacity of C6GKO-P25 cells to produce Th1-type cytokines in the lung parenchyma at either day 7 or 20 (Figure 6B). Therefore, the reduction in Th1 cytokine production in the effector stage of CD4^+^ T-lymphocyte responses in CXCR6-deficient mice (Figures 3D, 5D) was not due to an intrinsic property of CXCR6^KO^ CD4^+^ T-lymphocytes.

### Evaluation of the Retention and Function of Lung Resident Memory T-Lymphocytes in CXCR6-Deficient Mice

**Figure 7:**
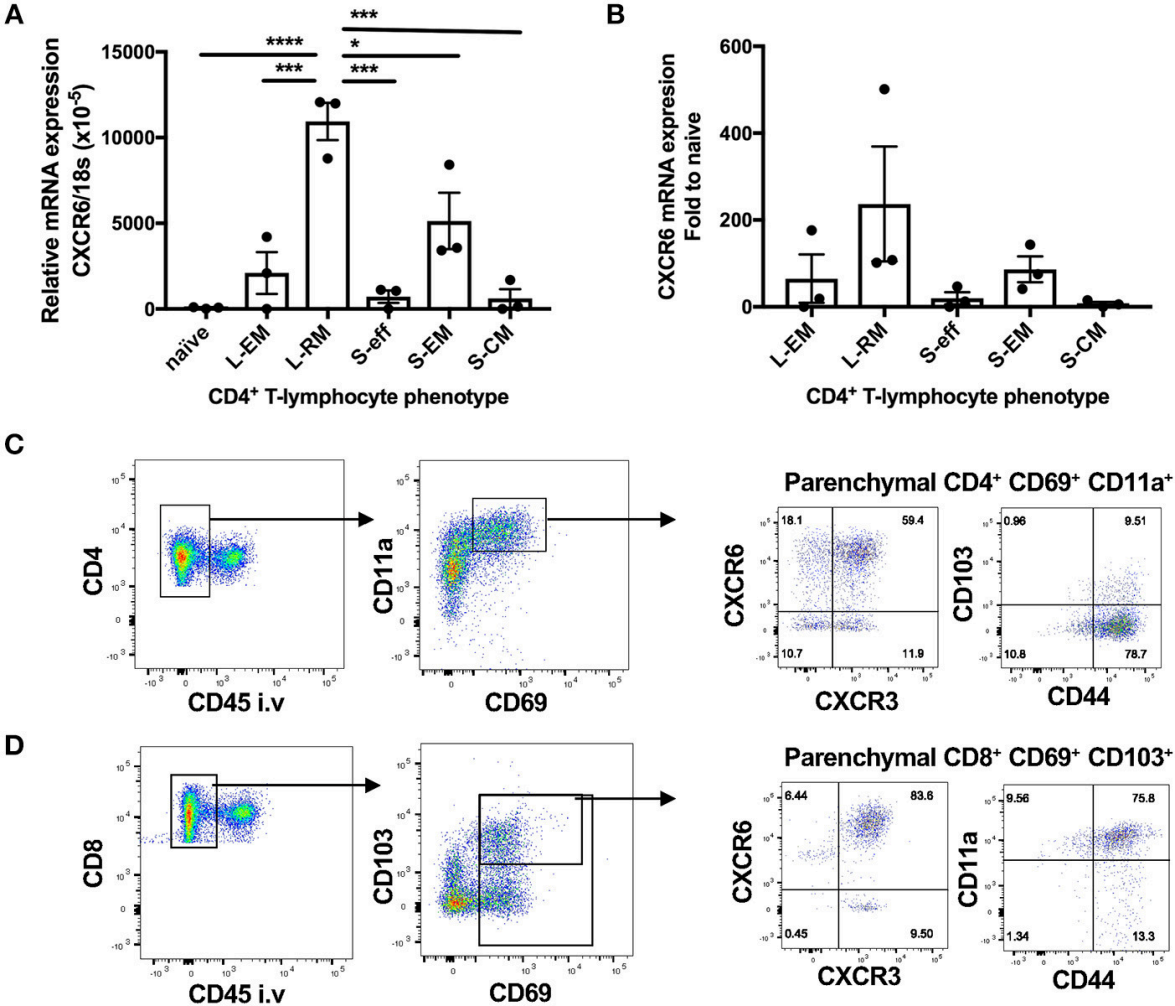
CXCR6 is highly expressed by lung resident memory T-lymphocytes after influenza infection. **(A,B)** 6 weeks following transfer of P25-specific CD45.1^+^ CD4^+^ T-lymphocytes (naïve) and infection with PR8-P25, transferred cells were purified from the lungs, and spleens by sorting according to memory phenotype: lung effector memory (L-EM, CD69^−^CD62L^−^), lung resident memory (L-RM, CD69^+^), spleen effector memory (S-EM, CD69^−^CD62L^−^), or spleen central memory (S-CM, CD69^−^CD62L^+^). Effector P25 cells (S-eff) were sorted at 11 days p.i. The transcriptional expression of CXCR6 was determined by RT-PCR. Data are the means ± SEM of three replicate experiments (total *n* = 20) and are shown as **(A)** CXCR6 expression relative to 18S mRNA and **(B)** fold change in CXCR6 expression compared to naïve cells. The statistical significance of differences were analyzed by ANOVA with Bonferroni *post-hoc* comparison (^*^p < 0.05, ^***^p < 0.001, ^****^p < 0.0001). **(C,D)** CXCR6-reporter mice (*n* = 5) were infected i.n with PR8-P25 and at 6 weeks were injected i.v with anti-CD45 antibody to label intra-vascular leukocytes. Memory T-lymphocytes were identified by flow cytometry. Identification of **(C)** CD4^+^ T_RM_ [parenchymal (CD45^−^) CD69^+^ CD11a^+^] and **(D)** CD8^+^ T_RM_ [parenchymal (CD45^−^) CD69^+^ CD103^+^]. Expression of CXCR3, CXCR6, CD44, and CD103 are shown. Data are representative of repeat experiments.

**Figure 8:**
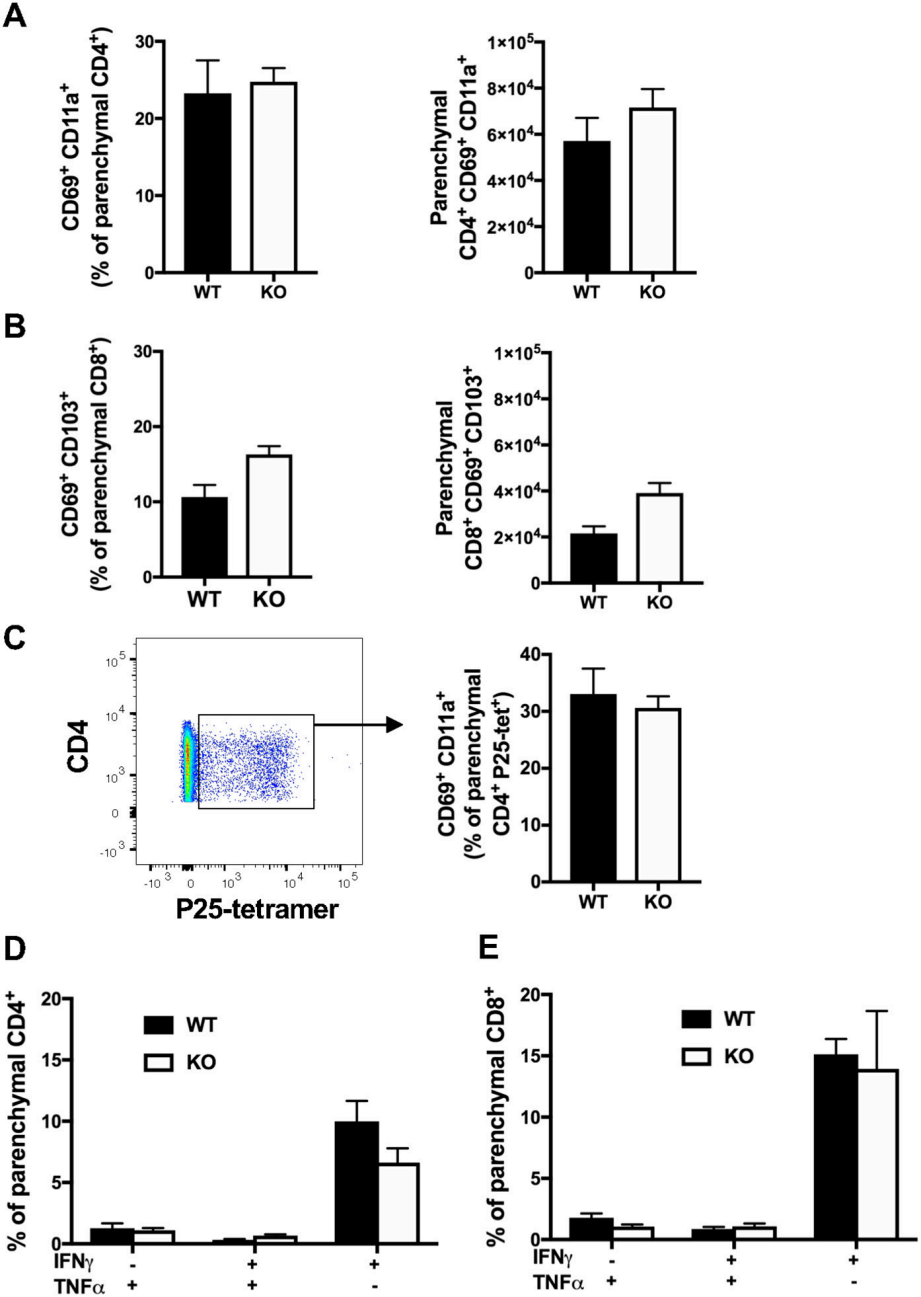
CXCR6-deficiency does not impair the retention or function of tissue-resident T-lymphocytes in the lungs at 6 weeks. CXCR6^WT^ or CXCR6^KO^ mice (*n* = 5) were infected i.n with PR8-P25 and at 6 weeks were injected i.v with anti-CD45 antibody to label intra-vascular leukocytes. Endogenous memory T-lymphocytes were identified by flow cytometry. The proportion and number of total **(A)** CD4^+^ T_RM_ [parenchymal (CD45^−^) CD69^+^ CD11a^+^] and **(B)** CD8^+^ T_RM_ [parenchymal (CD45^−^) CD69^+^ CD103^+^]. **(C)** Proportion of P25-specific CD4^+^ T_RM_ [parenchymal (CD45-) CD4^+^ P25-tetramer^+^ CD69^+^ CD11a^+^] were identified by tetramer staining. Lung leukocytes were incubated with **(D)** P25 peptide to stimulate memory CD4^+^ T-lymphocytes or **(E)** NP_366−375_ peptide to stimulate memory CD8^+^ T-lymphocytes, followed by ICS and flow cytometry to assess cytokine production by parenchymal cells (CD45^−^). Data are the means ± SEM and are representative of repeat experiments. The statistical significance of differences were analyzed by ANOVA with Bonferroni *post-hoc* comparison.

CXCR6-deficiency is known to result in reduced T_RM_ responses in the skin and liver (2, 14–16). To investigate whether CXCR6 is also preferentially expressed by memory T-lymphocytes in the lungs and if it is essential for the retention and function of T_RM_, retention of lung T_RM_ was examined 6 weeks after PR8-P25 infection. At this time point, there is no viral antigen remaining in the lungs and the majority of T-lymphocytes remaining are memory rather than effector phenotype (30). Adoptively transferred, P25-specific CD4^+^ T-lymphocytes were sorted from the lungs and spleens of WT mice p.i according to their memory phenotype, and the transcriptional expression of CXCR6 was determined by RT-PCR. Naïve P25-specific CD4^+^ T-lymphocytes had very low expression of CXCR6 mRNA, and this was upregulated in both lung and spleen effector cells. Strikingly, lung resident memory cells had highly significant upregulation of CXCR6 mRNA compared to other memory populations (Figures 7A,B). In addition, endogenous CD4^+^ and CD8^+^ T_RM_ were enumerated by flow cytometry in CXCR6-reporter mice. The majority of CD4^+^ T_RM_, that were defined as CD69^+^ CD11a^+^ parenchymal CD4^+^ T-lymphocytes, expressed CXCR6, and also CXCR3, but not CD103 (Figure 7C). In addition, the majority of CD8^+^ T_RM_, that were defined as CD69^+^ CD103^+^ parenchymal CD8^+^ T-lymphocytes, expressed CXCR6, CXCR3, and CD11a (Figure 7D). Despite the high level of CXCR6 expression by these populations however, in CXCR6-deficient mice there was no reduction in either the proportion or number of total CD4^+^ and CD8^+^ T_RM_ in the lungs (Figures 8A,B). To confirm antigen-specific CD4^+^ T_RM_ generated in response to PR8-P25 infection were not affected by CXCR6-deficiency, P25-tetramer positive T_RM_ were also enumerated, and similarly, there was no reduction in this population in CXCR6-deficient mice (Figure 8C). To assess the cytokine producing capacity of these T-lymphocytes, leukocytes from the lungs were recalled with peptides from rIAV-P25. There was no change in cytokine production by P25-specific CD4^+^ T-lymphocytes (Figure 8D) or NP-specific CD8^+^ T-lymphocytes (Figure 8E) in CXCR6-deficient mice compared to WT mice. Therefore, CXCR6 is highly expressed by lung memory T-lymphocytes, but is not required for the retention and function of CD4^+^ or CD8^+^ T_RM_ following influenza infection.

## Discussion

Understanding the factors that regulate protective leukocyte function in the lungs during pulmonary infection is essential for the development of more effective vaccines and therapies. Chemokine receptors play an important role in the recruitment and retention of T-lymphocytes into non-lymphoid tissues. CXCL16 is constitutively expressed by human bronchial epithelia (10) and also by activated alveolar macrophages (17), suggesting it may contribute to the recruitment or retention of T-lymphocytes in the lungs. Previous studies with adenovirus-based TB vaccines suggested there was a correlation between lung CXCR6^+^ CD8^+^ T-lymphocytes induced by pulmonary vaccination and protection against *M. tuberculosis* in mice (7, 25), but an essential role for CXCR6 in protective responses to pulmonary infections has not been demonstrated.

Contrary to expectation, CXCR6-deficiency was advantageous in protection against both pathogens. In *M. tuberculosis* infection, there was no change to the bacterial burden at an early time point of 3 weeks. However, by 6 and 12 weeks there was a reduction in the lung bacterial burden in CXCR6-deficient mice (Figure 2A), but no difference in the liver or spleen. CXCR6 is highly upregulated on T-lymphocytes in the liver during *Listeria monocytogenes* infection, possibly in response to CXCL16 expressed by liver sinusoidal endothelial cells. Nevertheless, CXCR6-deficiency did not impair control of *L. monocytogenes*, but rather reduced bacterial burden in the liver (14). CXCR6-deficient mice also showed reduced effects of rIAV infection, with decreased weight loss and earlier recovery (Figure 4A). Similar resistance phenotypes were observed in CXCR3-deficient mice (34). As with CXCR6, CXCR3 is expressed by effector Th1 and CD8^+^ T-lymphocytes, NK, and NKT cells, and is upregulated by these cells in the lungs during IAV infection (34). CXCR3-deficient mice on a BALB/c background had improved control of *M. tuberculosis* in the lungs at chronic stages of infection (35). CXCR3-deficiency also rescued CCR5-deficient mice from IAV-induced mortality, with CXCR3 and CCR5 double-deficiency reducing excessive inflammatory infiltrate to the lungs, although notably without impairing the generation of T-lymphocyte mediated protective memory (34).

The immune response to *M. tuberculosis* infection is immensely complex, but importantly, multiple studies have demonstrated that T-lymphocytes are indispensable for protection (36, 37). Early work in experimental mouse models demonstrated that T-lymphocytes were required for anti-*M. tuberculosis* immunity and that CD4^+^ T-lymphocytes were the primary mediators of this protection, shown both in transfer models (38–40) and in gene-deficient mice (41, 42). This has also been exemplified in humans by the observation that loss of CD4^+^ T-lymphocytes during HIV infection, or during corticosteroid treatment, increases susceptibility to mycobacterial diseases (43–45). It is also of interest that recent human trials examining BCG revaccination or *M. tuberculosis* protein subunit vaccines H4/IC31 or M72/AS01E, all of which predominantly stimulate CD4^+^ T- lymphocyte responses, demonstrated a significant level of protection against sustained infection (46, 47). While these CD4^+^ T-lymphocyte responses are essential, the importance of regulating these responses cannot be understated. This has been elegantly demonstrated in an examination of the role of inhibitory factors such as PD-1 and mitochondrial cyclophilin D expression, in preventing excessive T-lymphocyte responses that are detrimental to host defense (48, 49).

Given the critical role for lung T-lymphocyte responses in the control of *M. tuberculosis*, and also their requirement for viral clearance in IAV infection (50, 51), we had a particular focus on assessing the influence of CXCR6 on these populations. In the lungs of naïve CXCR6-reporter mice, small proportions of CD4^+^, and CD8^+^ T-lymphocytes and NK1.1^+^ cells expressed CXCR6, and this did not preferentially locate cells to the lung parenchyma or vasculature (Figures 1A,B). However, CXCR6 was substantially upregulated following pulmonary infection, particularly on T-lymphocytes (Figures 1D,F). In light of this, we hypothesized that deleting the receptor would reduce T-lymphocyte recruitment to the lungs. However, in both *M. tuberculosis* and IAV infection, CXCR6-deficiency did not impair the recruitment of T-lymphocytes to the lungs (Figures 3A, 5A), nor was there a consistent difference in expression of surface activation markers on effector cells (Figures 3B,C, 5B,C). Of note, CXCR3 was expressed on the majority of T-lymphocytes, consistent with previous reports that this is upregulated on lung-homing T-lymphocytes, or Th1-type CD4^+^ T-lymphocytes (34, 52). Adoptive transfer of CXCR6-deficient and WT P25-specific CD4^+^ T-lymphocytes showed an increase of CXCR6-deficient cells in the lungs and BAL at day 7 after PR8-P25 infection (Figure 6A), but by day 20 equivalent numbers of WT and CXCR6-deficient cells were present at all sites (Figure 6A). This implies that in the absence of CXCR6 expression, CD4^+^ T-lymphocytes may be recruited to the lungs earlier or potentially undergo a greater degree of proliferation at the site of infection.

Early reports on CXCR6 expression by T-lymphocytes indicated that IL-12 enhanced expression of CXCR6 on CD4^+^ T-lymphocytes, while IL-4 inhibited it. This suggested that CXCR6 expression defines type 1-polarized T-lymphocytes that home to non-lymphoid tissues (4). In response to *M. tuberculosis* or rIAV, CXCR6^KO^ mice developed Th1 cells, but at the peak of the effector response in both infections, there were reduced numbers of lung Th1 cells secreting IFNγ and TNFα (Figures 3D, 5D). Therefore, although CXCR6 is expressed by Th1 or Tc1-type cells, CXCR6 is not exclusive to these cells (4, 52) and is not required for T-cell polarization.

In the case of *M. tuberculosis* infection, the reduced Th1-response observed in CXCR6-deficient mice at 6 weeks could be related to the decreased bacterial load in the lungs, but there was no corresponding reduction in cytokine responses by TB10.4_3−11_-specific CD8^+^ T-lymphocytes in the lung parenchyma, nor were there reduced systemic Th1 or CD8^+^ T-lymphocyte responses. This suggests that the reduced Th1-response observed was not simply caused by a reduced bacterial burden alone. It would be of interest to study Th1-cytokine responses in the lungs of CXCR6-deficient mice to a wider range of mycobacterial antigens, such as culture filtrate protein. In the lungs of CXCR6-deficient mice, there was no significant change in NP copy number following rIAV infection (Figure 4B), nor in cytokine production by NP-specific CD8^+^ T-lymphocytes (Figures 5F,G). Therefore, it is unlikely that reduced viral load in CXCR6-deficient mice contributed to the reduced Th1-response in the lungs. Following adoptive transfer of CXCR6-deficient or WT P25-specific CD4^+^ T-lymphocytes and rIAV infection, there was no difference in the production of Th1-type cytokines by P25-specific T-lymphocytes in the lung parenchyma (Figure 6B). Therefore, the reduced Th1-response in the lungs of rIAV-infected CXCR6-deficient mice was not an intrinsic property of the CD4^+^ T-lymphocytes, but rather a result of the integrated immune response of the host to the pathogen.

Pulmonary vaccination with both BCG and PR8-P25 induces T_RM_ in the lung parenchyma, and these lung resident T-cells can confer protection against *M. tuberculosis* in the absence of circulating memory cells (30, 53). The recent findings that CXCR6 is highly upregulated on long term memory CD8^+^ T-lymphocytes in human BAL and lungs (21–23) suggest a role for CXCR6 in retention of T-lymphocytes at the site. In addition, CXCR6 contributes to the formation of T_RM_ in the liver and skin (2, 14–16). However, at 6 weeks after rIAV infection, there was no deficiency in memory CD4^+^ or CD8^+^ T-lymphocytes with a T_RM_ phenotype in CXCR6-deficient animals (Figures 8A,B,C), nor in the capacity of T_RM_ to secrete cytokines (Figures 8D,E). Together these data indicate that while CXCR6 may be highly expressed by T_RM_ in the lungs (Figure 7), it is not required for their formation or function. It is possible however that CXCR6-deficiency alters the kinetics of T-lymphocyte recruitment to the lungs early in infection or influences T-lymphocyte positioning within the parenchyma. Further, regulating the spatial compartmentalization of pro- and anti-inflammatory signals within the local context of the granuloma in mycobacterial infection may be critical for controlling bacterial dissemination (54, 55). Examining the impact of CXCR6-deficiency on the spatial organization within the granuloma is of interest for future investigation.

CXCR6 is expressed by other lymphoid cells, such as NK and NKT cells and ILCs. In the absence of CXCR6, the distribution of NKT cells was altered, with a decrease in the lungs and liver of naïve mice and a corresponding increase in the bone marrow (56). CXCR6 was required for the maintenance of these populations, but not their function, suggesting that CXCR6 may not directly promote survival but directs cells to sites where survival signals are available (57, 58). The cytokine profile of antigen-stimulated NKT cells is regulated by CXCR6-CXCL16 interaction, with reduced IFNγ production in the absence of CXCL16 signaling (56, 59). It is possible that reduced IFNγ production by NK/NKT cells led to reduced CD4^+^ Th1-cell differentiation, and therefore decreased IFNγ responses by P25-specific effector CD4^+^ T-lymphocytes in the lungs (Figures 3D, 5D). In the absence of CXCR6, regulatory functions of NKT cells are also disrupted, one study finding that this led to aggravated autoimmune kidney inflammatory disease (60). In our study, a proportion of NK1.1^+^ cells expressed CXCR6 in naïve reporter mice (Figure 1B). In naive CXCR6-deficient mice, there was a small but non-significant reduction in the NK1.1^+^ population in the lungs compared to WT mice (Figure 1C), and there was no change to the distribution of NK1.1^+^ cells between the parenchyma or vasculature (data not shown). CXCR6 expression on NK1.1^+^ cells was upregulated following infection, but there was no difference in the number of these cells between CXCR6-deficient and WT mice (Figures 3A, 5A). This is consistent with previous studies demonstrating that during inflammation, other chemokine receptors, such as CCR4, are sufficient for NKT recruitment to the lungs (61). CXCR6 has also been reported to be required for the maintenance of ILC3s in the intestine (56, 62), and in CXCR6-deficient mice these cells were retained in the bone marrow (63). It is possible CXCR6 plays a similar role at other mucosal surfaces, such as the lung, and this should be investigated in future work.

In summary, CXCR6-deficiency was associated with increased protection against *M. tuberculosis* in the lungs at 6-weeks and reduced systemic effects of acute rIAV infection, determined by reduced weight loss, without changing T-lymphocyte recruitment to the lungs. There was a reduction in antigen-specific Th1-cytokine production acutely after *M. tuberculosis* and rIAV, but Th1 polarization at later time points was not affected. Earlier studies have suggested CXCR6 or CXCL16 as possible therapeutic targets in pulmonary inflammatory diseases, to reduce the influx of T-lymphocytes contributing to immunopathology (4, 17, 18). However, in our study, although CXCR6 was strongly upregulated on both effector and memory T-lymphocytes, the receptor was redundant for T-lymphocyte recruitment. Alternative receptors, such as CXCR3, CCR5, and CCR4, may compensate to recruit T-lymphocytes to the lungs. Moreover, CXCR6 was not required for retention of memory T-lymphocytes in the lungs and so may not be a reliable marker of protective T_RM_ in the lungs nor a correlate of protection. Therefore, although CXCR6 contributes together with other chemokine receptors to the integrated T-lymphocyte response to *M. tuberculosis* and influenza infection, it is not essential for the control of these infections. Dissecting the role of chemokine receptors in pulmonary inflammation may further our understanding of T-lymphocyte responses to respiratory pathogens and aid the development of preventative or therapeutic strategies.

## Author Contributions

AA, MF, JS, and WB conceived the project, designed the experiments and analyzed and interpreted the data. AA, MF, LL, DQ, EA, and SS conducted the experiments and analyzed the data. JS provided the recombinant influenza virus utilized. AA and WB wrote the manuscript, which was reviewed and approved by all the authors.

### Conflict of Interest Statement

The authors declare that the research was conducted in the absence of any commercial or financial relationships that could be construed as a potential conflict of interest.
