# Supplementary material for: CXCR6-Deficiency Improves the Control of Pulmonary Mycobacterium tuberculosis and Influenza Infection Independent of T-Lymphocyte Recruitment to the Lungs
Source: Front Immunol. 2019 Mar 7;10:339. doi: 10.3389/fimmu.2019.00339 (PMC6416161; doi:10.3389/fimmu.2019.00339)
Supplement: Supplementary file 1 [file Data_Sheet_1.pdf]

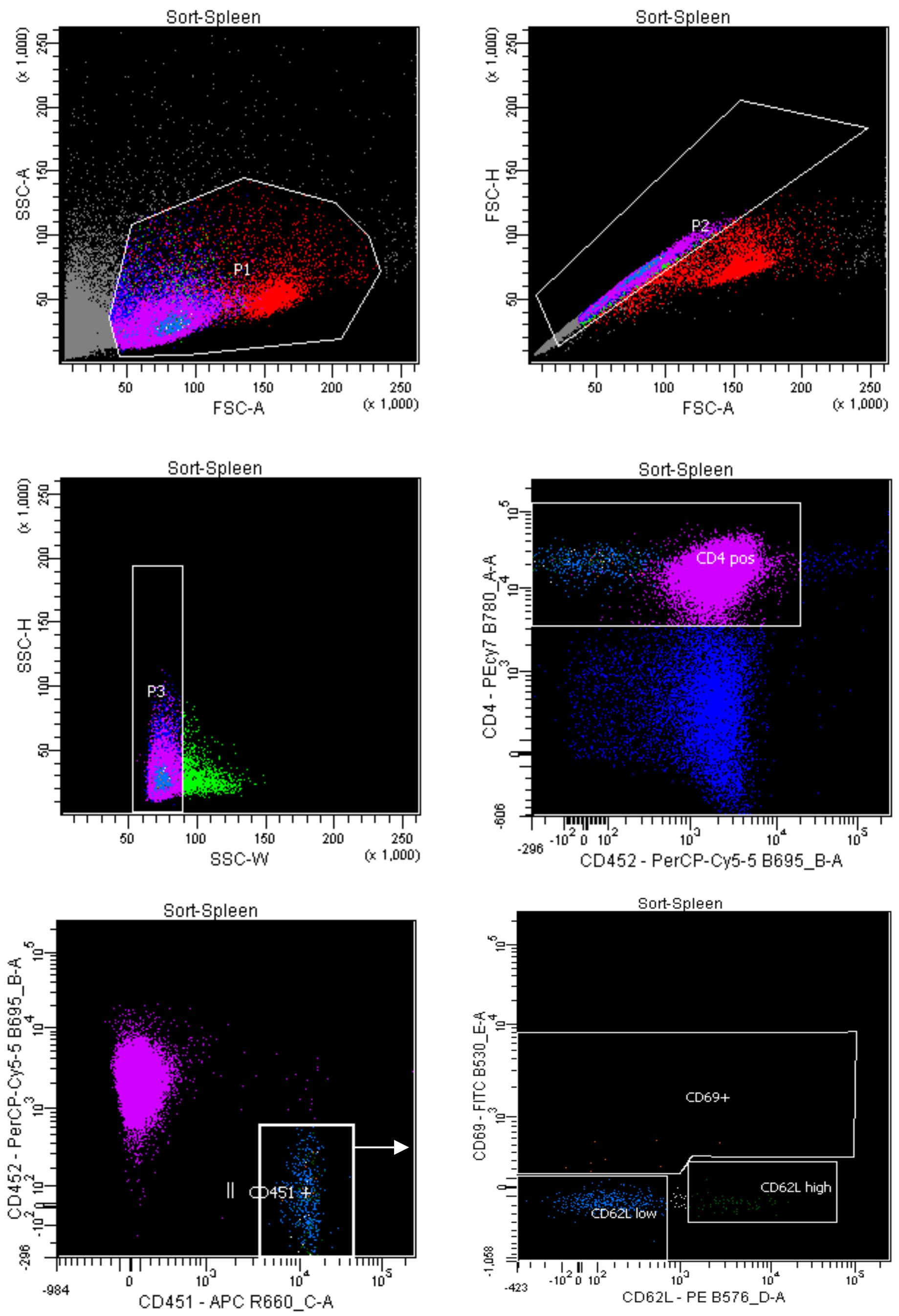

Supplementary Figure 1. Gating strategy for sorting of memory P25-specific T-lymphocytes from the spleen. 6 weeks following transfer of P25-specific CD45.1$^+$ CD4$^+$ T-lymphocytes (naïve) and intra-nasal infection with PR8-P25, transferred cells were purified from the spleens by sorting according to memory phenotype: spleen effector memory (S-EM, CD69$^-$CD62L$^-$) or spleen central memory (S-CM, CD69$^-$CD62L$^+$). Effector P25 cells (S-eff) were sorted at 11 days p.i (CD4$^+$ CD45.1$^+$).

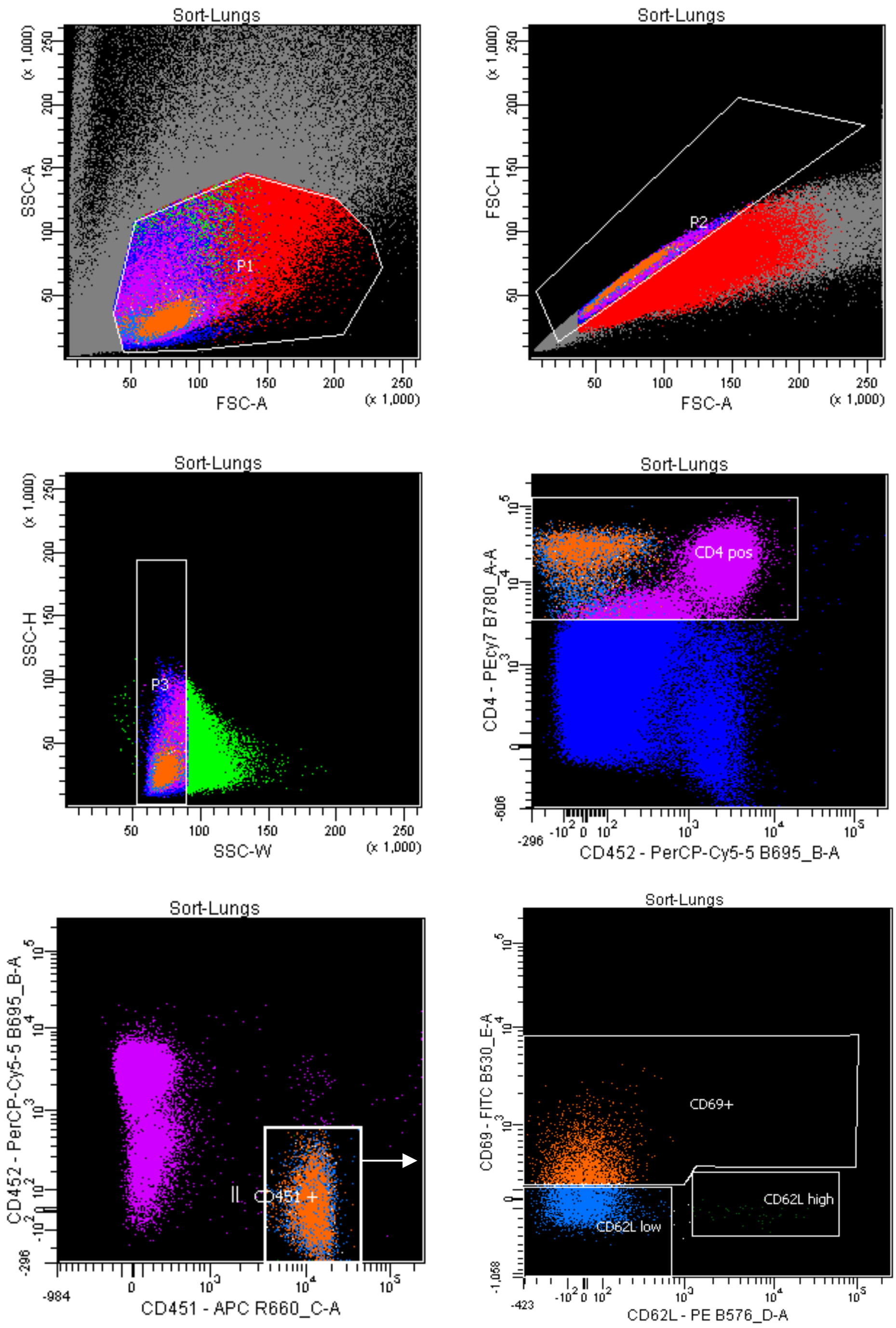

Supplementary Figure 2. Gating strategy for sorting of memory P25-specific T-lymphocytes from the lungs. 6 weeks following transfer of P25-specific $CD45.1^+$ $CD4^+$ T-lymphocytes (naïve) and intra-nasal infection with PR8-P25, transferred cells were purified from the lungs by sorting according to memory phenotype: lung effector memory (L-EM, $CD69^-CD62L^-$) or lung resident memory (L-RM, $CD69^+$).

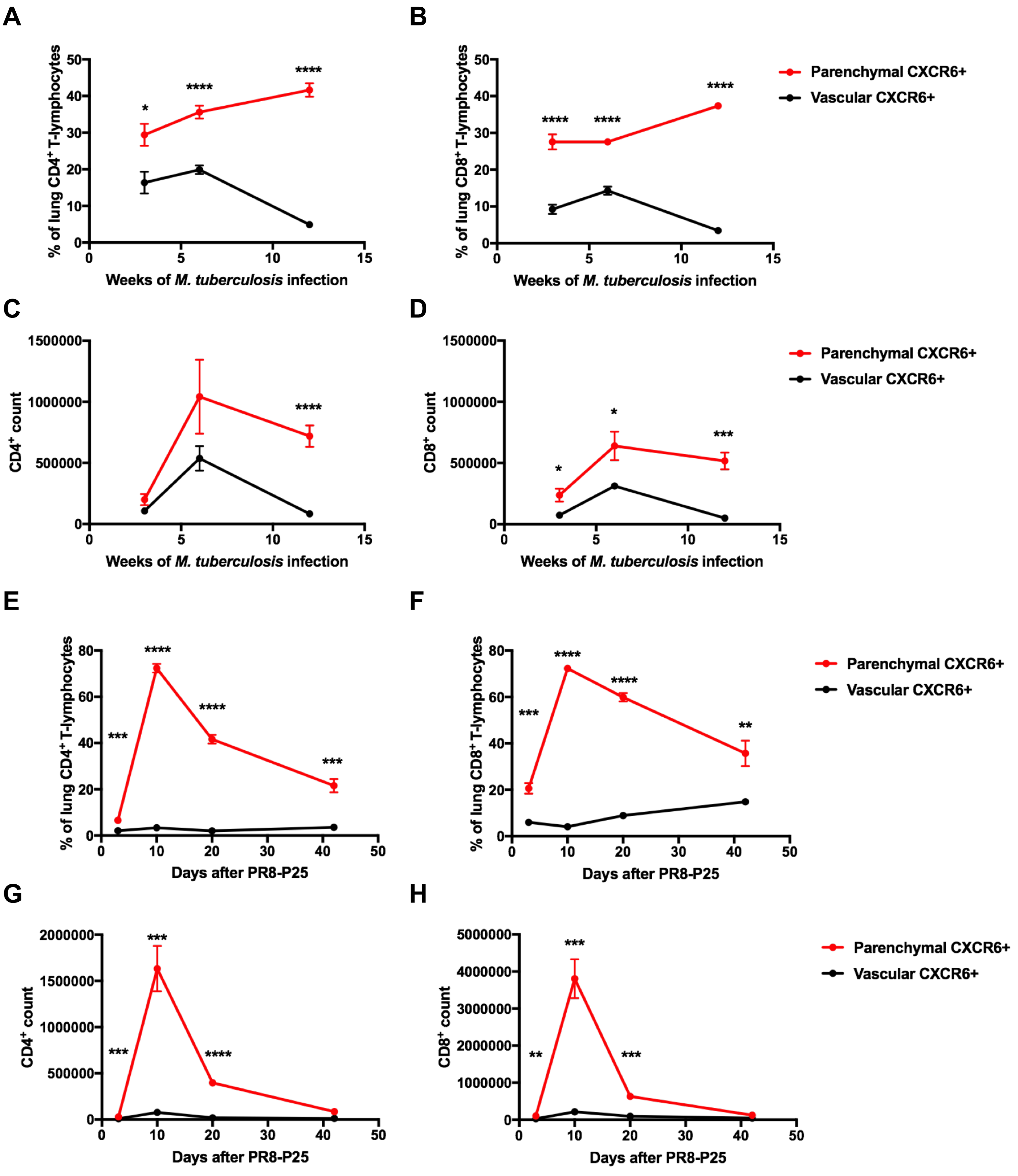

Supplementary Figure 3. Kinetics of lung CXCR6⁺ CD4⁺ and CD8⁺ T-lymphocytes after pulmonary infection. CXCR6$^+$ CD4$^+$ and CD8$^+$ T-lymphocytes were quantitated in CXCR6-reporter mice in the lung parenchyma (red) or vasculature (black). Frequency of CXCR6$^+$ (**A**) CD4$^+$ and (**B**) CD8$^+$ T-lymphocytes, or number of CXCR6$^+$ (**C**) CD4$^+$ and (**D**) CD8$^+$ T-lymphocytes at 3, 6 or 12 weeks after *M. tuberculosis* infection (n=5). Frequency of CXCR6$^+$ (**E**) CD4$^+$ and (**F**) CD8$^+$ T-lymphocytes, or number of CXCR6$^+$ (**G**) CD4$^+$ and (**H**) CD8$^+$ T-lymphocytes at 3, 10, 20 or 42 days after PR8-P25 infection (n=4-5). Data are the means ± SEM. The statistical significance of differences between parenchymal and vascular cells at each time point were analysed by multiple t tests with correction for multiple comparisons using the Holm-Sidak method (*$p<0.05$, **$p<0.01$, ***$p<0.001$, ****$p<0.0001$).

A

3 weeks

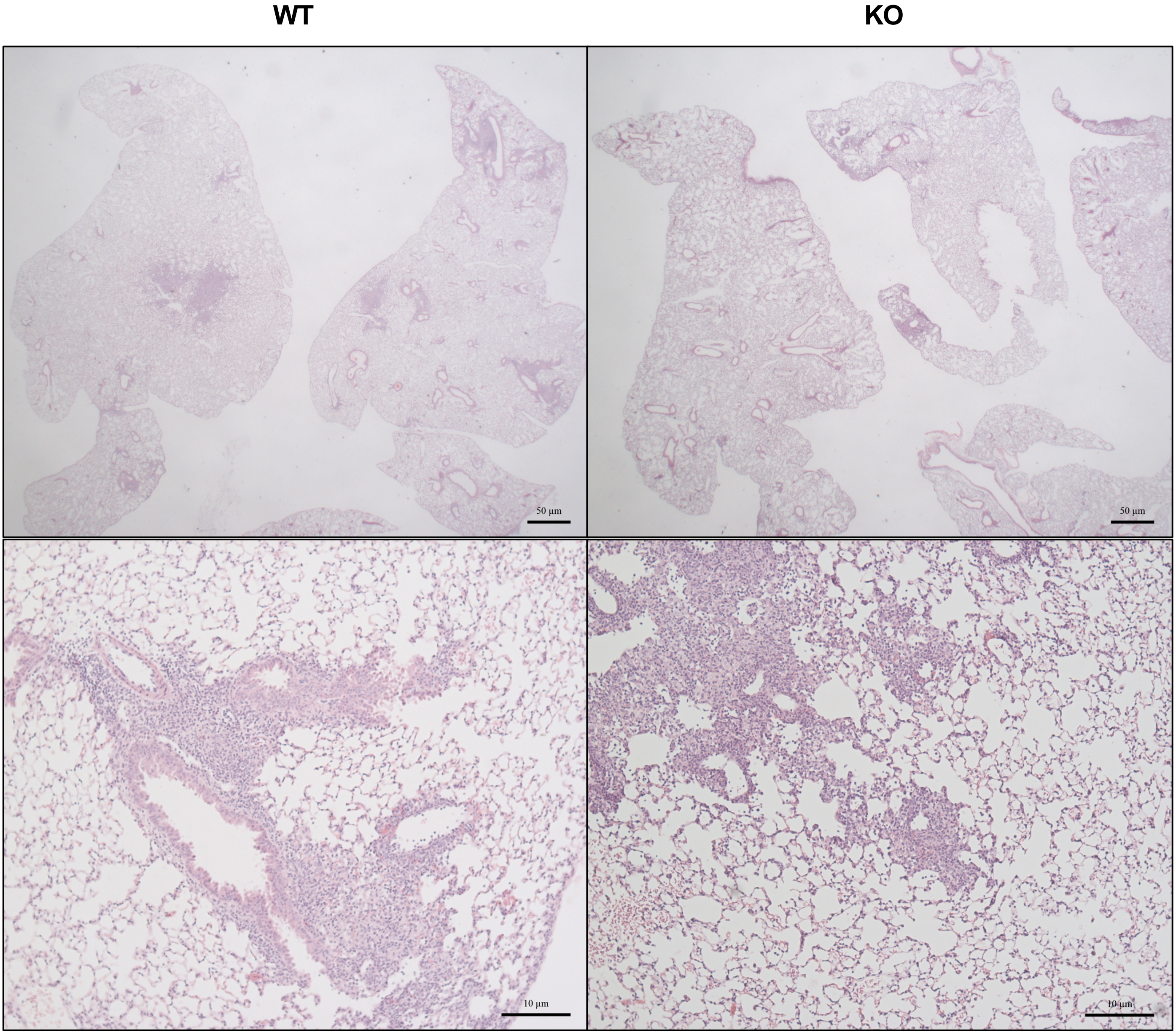

B

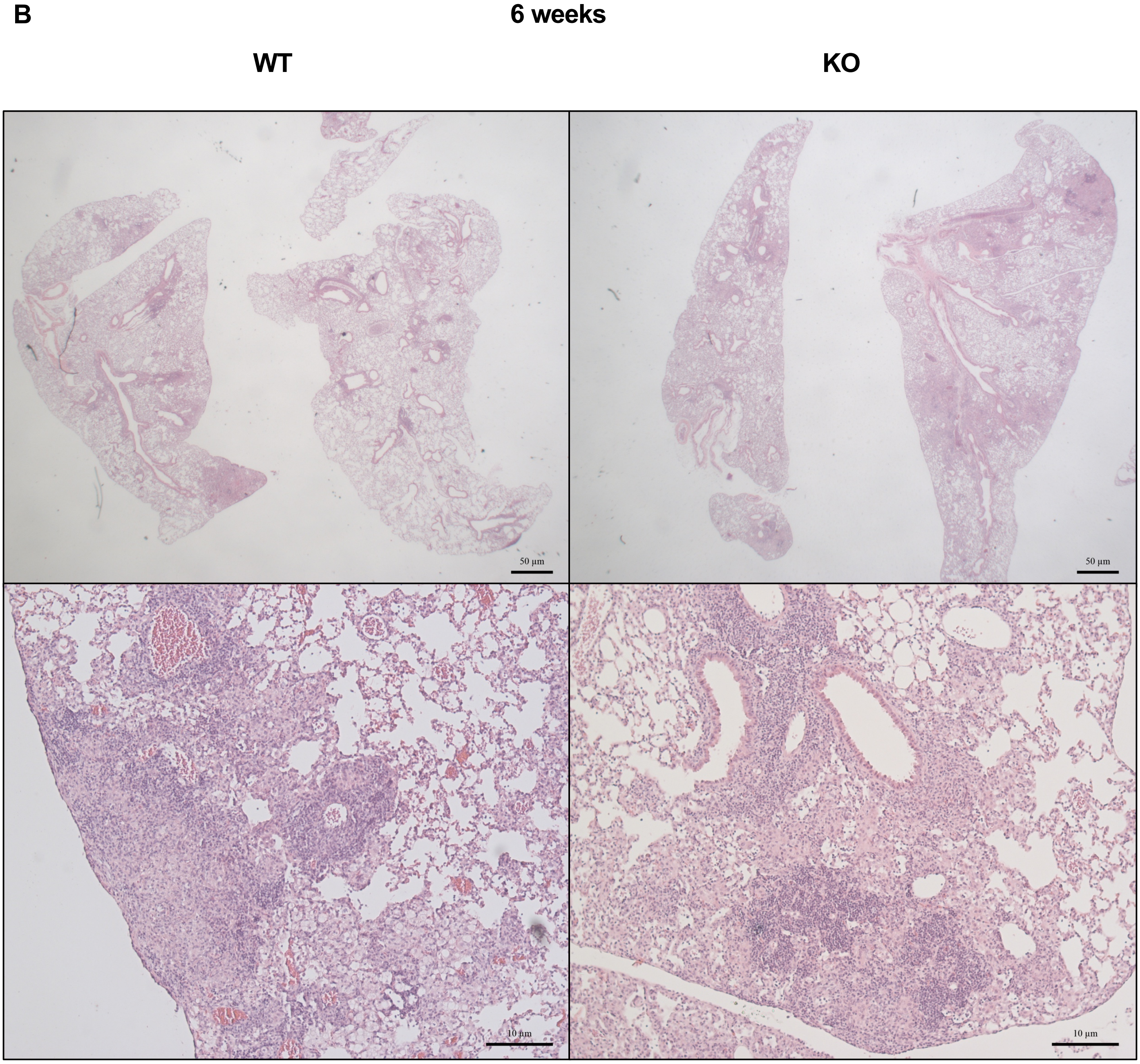

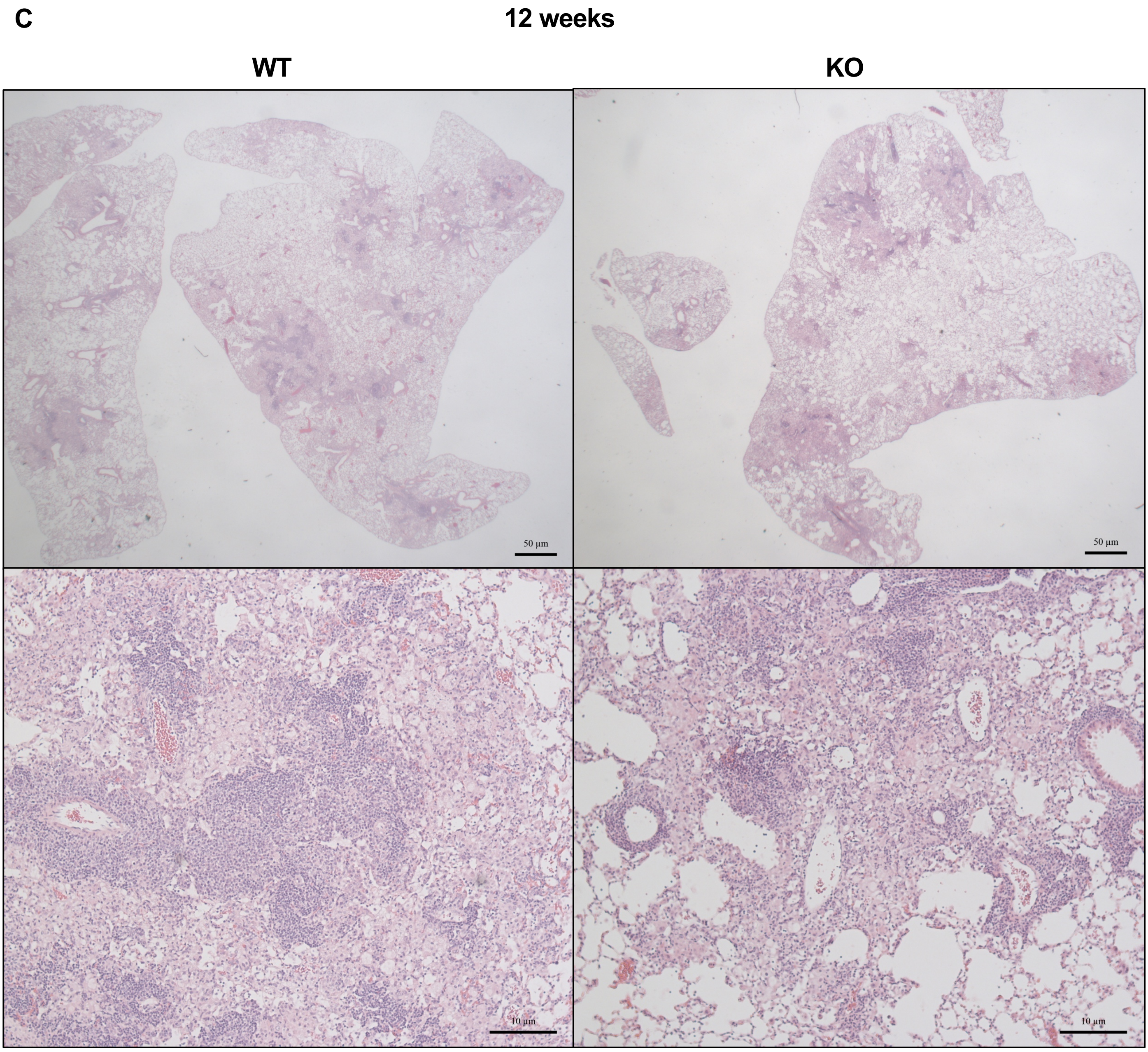

Supplementary Figure 4. Lung histology of CXCR6^{WT} and CXCR6^{KO} mice after *M. tuberculosis* infection. CXCR6^{WT} and CXCR6^{KO} mice (n=5) were infected with *M. tuberculosis* (100 CFU), and hematoxylin and eosin stained lung sections were examined at (**A**) 3 (**B**) 6 and (**C**) 12-weeks post infection. Representative images at 12.5x (top row, 500 µm scale bar) and 100x (bottom row, 100 µm scale bar) magnification are shown.

A **Day 3**

WT **KO**

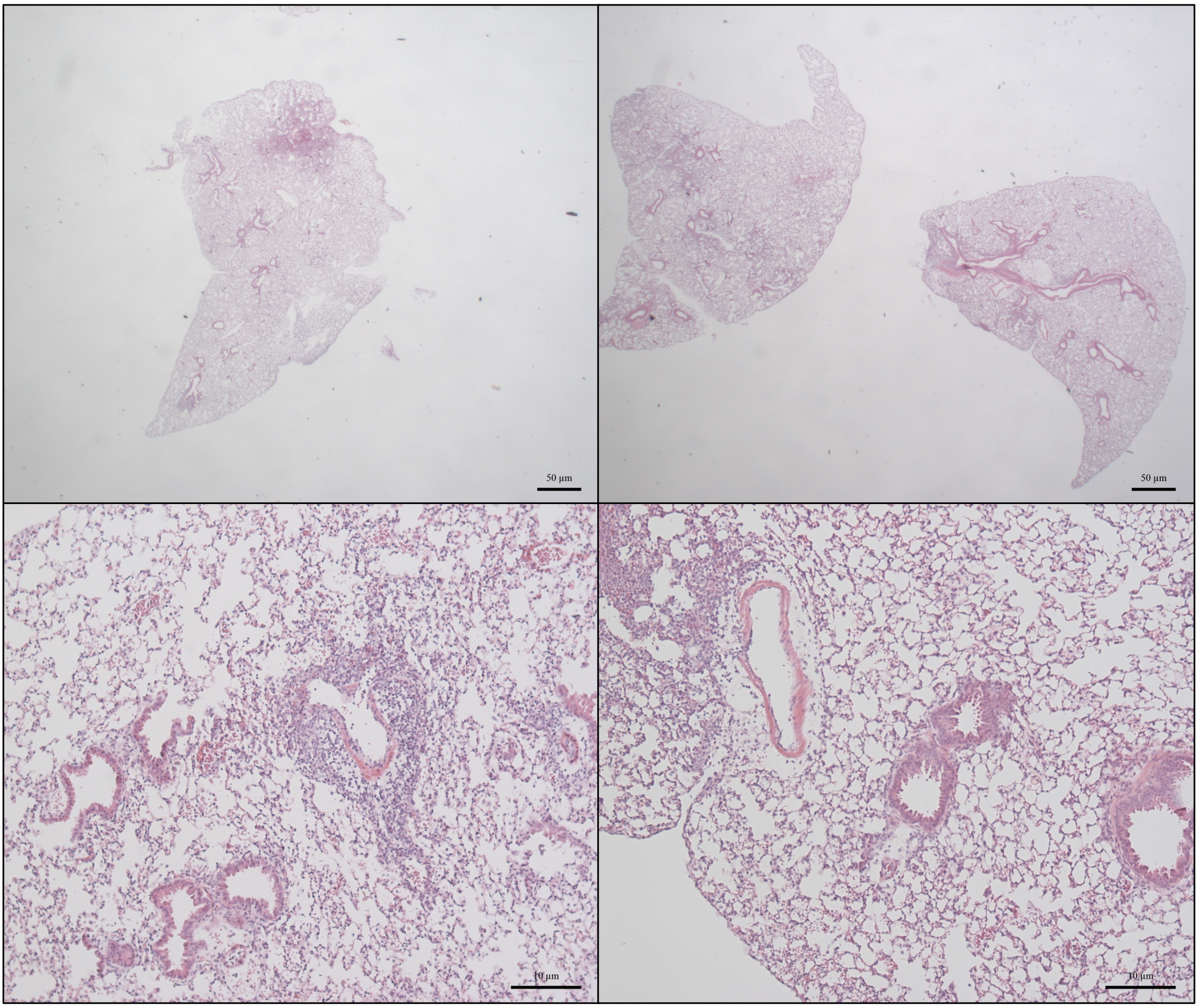

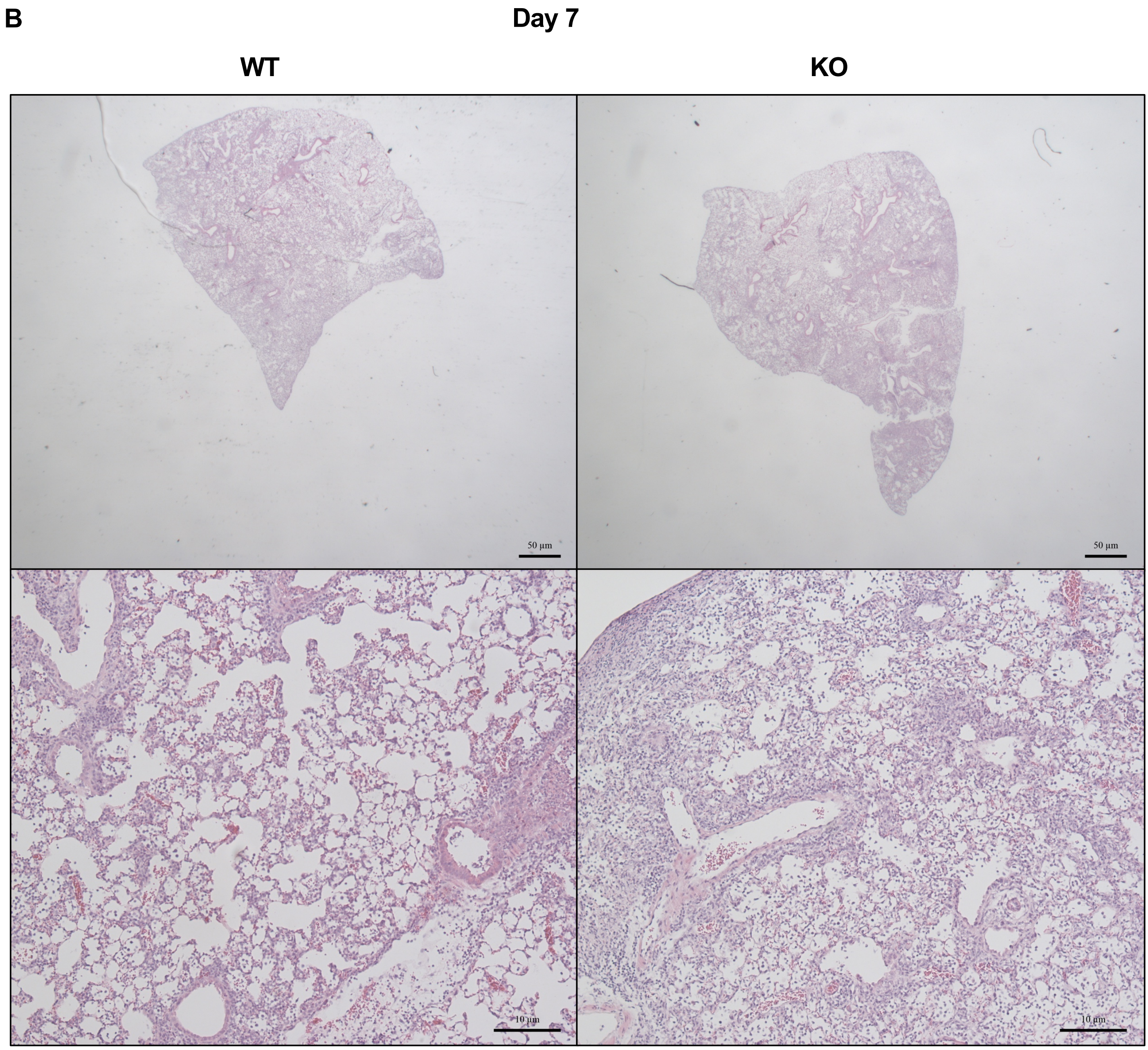

Supplementary Figure 5. Lung histology of CXCR6^{WT} and CXCR6^{KO} mice after PR8-P25 infection. CXCR6^{WT} and CXCR6^{KO} mice (n=3-5) were infected with PR8-P25 (20 PFU), and hematoxylin and eosin stained lung sections were examined at (**A**) 3 and (**B**) 7 days post infection. Representative images at 12.5x (top row, 500 µm scale bar) and 100x (bottom row, 100 µm scale bar) magnification are shown.
